# Understanding Racial HIV/STI Disparities in Black and White Men Who Have Sex with Men: A Multilevel Approach

**DOI:** 10.1371/journal.pone.0090514

**Published:** 2014-03-07

**Authors:** Patrick S. Sullivan, John Peterson, Eli S. Rosenberg, Colleen F. Kelley, Hannah Cooper, Adam Vaughan, Laura F. Salazar, Paula Frew, Gina Wingood, Ralph DiClemente, Carlos del Rio, Mark Mulligan, Travis H. Sanchez

**Affiliations:** 1 Department of Epidemiology, Rollins School of Public Health, Emory University, Atlanta, Georgia, United States of America; 2 Department of Psychology, Georgia State University, Atlanta, Georgia, United States of America; 3 Department of Infectious Diseases, Emory University School of Medicine, Atlanta, Georgia, United States of America; 4 Department of Behavioral Sciences and Health Education, Rollins School of Public Health, Emory University, Atlanta, Georgia, United States of America; 5 Institute of Public Health, Georgia State University, Atlanta, Georgia, United States of America; 6 Hubert Department of Global Health, Rollins School of Public Health, Emory University, Atlanta, Georgia, United States of America; Infectious Disease Service, United States of America

## Abstract

**Background:**

The reasons for black/white disparities in HIV epidemics among men who have sex with men have puzzled researchers for decades. Understanding reasons for these disparities requires looking beyond individual-level behavioral risk to a more comprehensive framework.

**Methods and Findings:**

From July 2010-Decemeber 2012, 803 men (454 black, 349 white) were recruited through venue-based and online sampling; consenting men were provided HIV and STI testing, completed a behavioral survey and a sex partner inventory, and provided place of residence for geocoding. HIV prevalence was higher among black (43%) versus white (13% MSM (prevalence ratio (PR) 3.3, 95% confidence interval (CI): 2.5–4.4). Among HIV-positive men, the median CD4 count was significantly lower for black (490 cells/µL) than white (577 cells/µL) MSM; there was no difference in the HIV RNA viral load by race. Black men were younger, more likely to be bisexual and unemployed, had less educational attainment, and reported fewer male sex partners, fewer unprotected anal sex partners, and less non-injection drug use. Black MSM were significantly more likely than white MSM to have rectal chlamydia and gonorrhea, were more likely to have racially concordant partnerships, more likely to have casual (one-time) partners, and less likely to discuss serostatus with partners. The census tracts where black MSM lived had higher rates of poverty and unemployment, and lower median income. They also had lower proportions of male-male households, lower male to female sex ratios, and lower HIV diagnosis rates.

**Conclusions:**

Among black and white MSM in Atlanta, disparities in HIV and STI prevalence by race are comparable to those observed nationally. We identified differences between black and white MSM at the individual, dyadic/sexual network, and community levels. The reasons for black/white disparities in HIV prevalence in Atlanta are complex, and will likely require a multilevel framework to understand comprehensively.

## Introduction

Since the beginning of the HIV epidemic, men who have sex with men (MSM) have been the predominantly affected risk group in the United States. Since the early 1990s, MSM have been the only US risk group for whom estimated HIV incidence has increased [1]. More recently, increases in incidence have been concentrated among young MSM of color [2]. Black MSM have over twice the prevalence of HIV than white men [3], [4], and data from HPTN 061 suggest that black MSM have HIV incidence rates over five times those of white MSM [5].

The reasons for these black/white disparities among MSM in HIV prevalence and incidence are unclear, but there is a consensus that differences in individual-level risk behaviors, such as frequency of unprotected anal intercourse (UAI) or numbers of sexual partners, do not account for the observed disparities [6]. Hypotheses that might explain black/white disparities in HIV prevalence include factors other than individual-level behaviors (for example, access to care, incarceration, and community burden of HIV), but some of these hypotheses have inadequate data thus far to conclude whether or not they are important. Similar racial disparities in prevalence and incidence of sexually-transmitted infections (STI) have been observed among MSM, and their causes are likewise not well understood [4], [6]. STI infections may also play a causal role in HIV acquisition risk and thus may contribute to the racial disparities in HIV infection [7], [8].

To address these scientific questions and to provide data to support tailored prevention packages for important subgroups of MSM, we developed “The Involvement” study, a multilevel cohort study of black and white MSM in Atlanta. We used Bronfrenbrenner's ecological systems model to conceptualize and study the individual, social and cultural influences contributing to the disparity in HIV infection between white and black MSM [9]. This theoretical framework supported the development of measures that capture individual-level determinants (Bronfrenbrenner's individual level: e.g., condom use, numbers of partners, substance use behaviors), sexual dyad and network determinants (Bronfrenbrenner's microsystem level: e.g., prevalence of HIV among partners, concurrency, transitivity) and community level determinants (Bronfrenbrenner's exosystem level: e.g., poverty rates, violent crime rates, and community HIV prevalence in the census tract of residence). Our goal is to understand how factors at these different levels operate together to give rise to (or perpetuate) the observed black/white disparities in HIV prevalence and incidence.

These same patterns of HIV and STI infection disparity exist among African American MSM in Atlanta, the city with the 8th highest rate of new HIV diagnoses in the country in 2011 [10]. MSM comprise the largest group living with HIV in Atlanta, and African American MSM are disproportionately affected, comprising about 60% of HIV-infected MSM whereas African Americans represent only about 30% of the overall Atlanta population [11]. Prevalent HIV infection in Atlanta is heterogeneous by geography, but is most concentrated in the center of the city as was illustrated by a previous study examining spatial clustering of HIV in Atlanta [11]. There are also approximately 60,000 new STI diagnoses in the state each year with approximately half occurring in Atlanta, and the majority of syphilis diagnoses are among African American men [12]. The size of the population of black MSM in Atlanta and the presence of a marked disparity in HIV infection among black MSM justifies exploring risk factors for HIV in Atlanta. This may be particularly important for understanding why this HIV infection disparity exists for black MSM in the US generally, because it is probable that the factors contributing to the disparity in Atlanta would be similar in other cities.

In this report we describe the methods used to recruit the cohort and the baseline data from over 800 black and white non-Hispanic MSM enrolled in Atlanta from 2010–2012. Our aim is to describe the characteristics of the cohort in terms of the multi-level framework described above, and to use baseline data from the cohort to describe differences in the levels of individual, sexual dyad and network, and community-level factors between enrolled black and white MSM.

## Methods

### Ethics Statement

This study was reviewed and approved by the institutional review boards of Emory University and Georgia State University.

### Sampling, Recruitment and Enrollment

InvolveMENt is an ongoing prospective cohort study at Emory University designed to examine the individual, dyadic, and community level factors that may contribute to the disparities in HIV and sexually transmitted infection prevalence and incidence between black and white MSM in Atlanta, Georgia. To adequately describe HIV incidence among these groups, the study was designed to enroll approximately equal numbers of HIV-negative black and white MSM into the prospective component of the study.

Recruitment occurred between June 2010 and December 2012. No sampling frame for MSM exists. We therefore used time-space venue sampling, supplemented by convenience sampling through Facebook. Venue sampling was used to choose a random sample of places where MSM congregate in Atlanta, according to the methods described for the National HIV Behavioral Surveillance System among MSM (NHBS-MSM) [13]. We began with the Atlanta venues sampling frame used in the 2008 round of NHBS-MSM and conducted additional formative research to explore the continued viability of these venues and additional venues for sampling. Briefly, all venues and day-time periods (VDTs) in which adequate numbers of MSM could potentially be sampled were included in a sampling frame. Sampling calendars for the month could also include up to 3 purposively selected VDTs. The types of venues that were included in the sampling frame included bars, dance clubs, fitness clubs or gymnasiums, Gay Pride events, parks, restaurants, retail businesses, sex establishments, social organizations, street locations, and other special events. Because of the higher baseline HIV prevalence among black MSM compared to white MSM and the desire to have a balanced prospective cohort, most purposively selected VDTs were those more likely to increase enrollment of black MSM. At the venues, study staff systematically approached men, used a brief recruitment script and administered screening questions. For Facebook sampling we placed paid banner advertisements through the Facebook advertising interface. Facebook advertisements were only delivered to men who were 18 years of age or older, reported currently residing in Atlanta, and who indicated an interest in men on their Facebook profiles. Men who clicked through the banner advertisement were screened for eligibility online; men who were eligible were called and invited to attend an in-person enrollment visit, at which time eligibility criteria were confirmed during informed consent.

Potentially eligible individuals were screened at the time of recruitment and at the enrollment visit. The goal of eligibility criteria was to produce a community-based sample of MSM at substantial risk for HIV infection. Individuals were eligible if they were male at birth, self-reported black or white race, could complete study instruments in English, currently lived in the Atlanta metropolitan statistical area, had at least 1 male sex partner in the previous 3 months and provided at least 2 means of contact. Men were excluded if they were of Hispanic/Latino ethnicity, had plans to move out of Atlanta in the next 2 years, were in a mutually monogamous relationship with a man, or were currently participating in any HIV prevention research study (enrollment for HPTN 061 occurred in Atlanta during this same time period). HIV status was not an eligibility criterion. During the recruitment period, two different age criteria were used. Initially, men ≥18 years were considered eligible; three months after enrollment began, eligible ages were additionally restricted to <40 years in response to the emerging consensus of the disproportionate burden of new HIV diagnoses among younger MSM [14].

After April 2011, some men eligible at recruitment were randomly offered screening for either InvolveMENt or another Emory University study of the sexual networks of MSM in Atlanta. Once men were offered screening for one study, if they were found to be ineligible or decided not to participate, they were not eligible to be screened for the other study. Those willing to participate were asked to provide detailed contact information for the purposes of scheduling the baseline enrollment visit at the study offices. At the baseline visit, for those eligible and agreeing to enroll, we obtained written, informed consent for all interview and specimen testing procedures. Participants were compensated $60 for the baseline visit.

### Survey Measures

At the baseline visit, participants completed an approximately 1.5-hour computer assisted self-interview questionnaire. The content was modified from instruments used in the first MSM cycle (2003–2005) of NHBS [15], a review of the literature, and qualitative evaluation of questionnaire modules. Domains included demographics, psychosocial scales, community characteristics, individual-level HIV-related behaviors, and a dyadic inventory of the most recent 5 sex partners in the previous 6 months (Figure S1) [16].

### Biomedical Measures

Regardless of self-reported HIV status, all participants were screened for antibodies to HIV with an FDA-approved HIV rapid test. Based on evolving availability of improved CLIA-waived rapid tests, men were screened with OraQuick (OraSure Technologies, Bethlehem, Pennsylvania) on oral mucosal transudate or blood, Clearview Complete (Alere, Waltham Massachusetts) on blood, or Insti (Bioanalytical, Richmond, British Columbia) on blood. For men who had a preliminary positive result on their HIV rapid test, additional specimens were collected by venipuncture for confirmatory western blot, CD4 and HIV viral load testing. Among those with preliminary positive results, confirmatory testing was by western blot; in one case, where additional specimens were not available for western blot testing, two additional HIV rapid tests were performed [17], [18]. Men who reported, either in the study questionnaire or to their HIV test counselor, that they had previously tested HIV-positive were considered to be aware of their HIV infection. All confirmatory, CD4, and viral load results were delivered to participants by phone, irrespective of self-reported HIV status. A staff specialist facilitated linkage to HIV care.

All participants were tested for syphilis and urethral *Neisseria gonorrhoeae* (GC) and *Chlamydia trachomatis* (CT). Beginning in October 2011, participants were also tested for rectal GC and CT using self-collected rectal swab specimens. Syphilis testing was conducted using the rapid plasma regain (RPR) test [19]; specimens that were reactive on the RPR test were reflexed to quantitative nontreponemal titers and treponemal IgG. New syphilis diagnoses were designated by experienced clinicians after reviewing all available data including previous RPR titers, if available, and treatment history. Testing for urethral and rectal GC and CT was by the Becton Dickinson ProbeTec ET *C. trachomatis* and *N. gonorrhoeae* Amplified DNA Assay (Sparks MD) [20]. The presence of TV was determined with Taq-Man PCR, using a test developed and validated at Emory which employs a homogenous kinetic polymerase chain reaction to amplify and detect a conserved part of a repeated DNA fragment of TV [21]. All participants who tested positive for an STI were notified and referred to a community treatment provider with treatment costs paid by the study.

All participants at baseline were also screened for biological markers of recent use of marijuana (THC), cocaine, morphine, amphetamines, and methamphetamines using a multi-drug screen dip card with a urine specimen (Alere, Waltham Massachusetts). Positive screening results were not confirmed. Separate consent was obtained for the collection and frozen storage of peripheral blood specimens that will support the exploration of supplementary and future hypotheses related to biological markers of HIV risk and infection among MSM. For participants who agreed to specimen storage and were HIV-positive at their second study visit, viral load testing on baseline visit specimens was performed to assess for primary HIV infection at enrollment. Primary infection was defined as having no previous HIV-positive test result, a negative HIV antibody screening test at the baseline visit, and a detectable HIV viral load test on the same day as the baseline negative HIV antibody screening test.

### Place-Based Measures

Participants' baseline home addresses were geocoded to their latitude/longitude and assigned to 2010 US Census Tracts. We were able to geocode 99.3% of all addresses. Clustering of census tracts including black and white participants was assessed using the Getis-Ord G* statistic. Tract characteristics were compiled from several administrative sources that past empirical and theoretical work suggested might be relevant to racial disparities among MSM. These include tract-level poverty rates, the percent of residents who identified as non-Hispanic black, the percent of households with a male same-sex couple, violent crime rates, the spatial density of off-premises alcohol outlets, and prevalence of persons living with HIV (data sources available in Table S1).

Geocoding was completed using 10.0 US Streets Geocode Service (ArcGIS Online. ESRI, Redlands, CA) and mapping in ArcGIS 10.3 (ESRI, Redlands, CA). To ensure confidentiality when these locations were mapped, we randomized points within the assigned census tract.

### Prospective Follow-Up

All participants confirmed to be HIV-negative at the baseline visit are followed prospectively for 2 years to observe HIV and STI incidence. Visits occur at 3, 6, 12, 18, and 24 months after baseline, and visit procedures for follow-up visits are nearly identical to the initial visit. Participants are compensated for each follow-up visit (up to $220 total). Study discontinuation occurs after the 24-month visit or HIV seroconversion. Accrual of follow-up time is projected to end in mid-2014.

### Analytical Methods

The recruitment, screening, and enrollment processes were summarized for black and white men. Men were excluded from analyses if they were later identified as duplicate enrollments (n = 6) or determined to be ineligible after enrollment (n = 2). Key demographic, sexual behavior, self-reported drug use, and HIV-testing characteristics were summarized descriptively and compared between black and white MSM participants using chi-square and *t*-tests. STI and HIV infection prevalence was calculated and compared by race using prevalence ratios (PR) with Farrington-Manning exact confidence-intervals (CI) [22]. Among HIV-positive participants, infection awareness, engagement in HIV care, mean CD4 count, and mean log_10_ viral load, were computed and compared by race using chi-square and *t*-tests.

The dyads described by participants were summarized separately, stratified by the participant's race. Among dyads, racial concordance, partner type and sex frequency, online meeting location, cohabitation, pre-sexual serodiscussion, and perceived HIV seroconcordance were tallied and compared by participant race using chi-square tests. Age concordance was compared by participant race using Lin's concordance coefficient, which measures adherence between two continuous values to the identity line and has values that range from -1 to 1, with interpretation similar to that of ordinary correlation coefficients [23], [24]. For the continuous characteristics of the census tracts where participants lived, means were computed and compared using *t*-tests. In any comparison where an expected cell had <5 observations, Fisher's Exact Test was used. Associations were considered significant at the alpha  = 0.05 level.

## Results

### Enrollment

**Figure 1:**
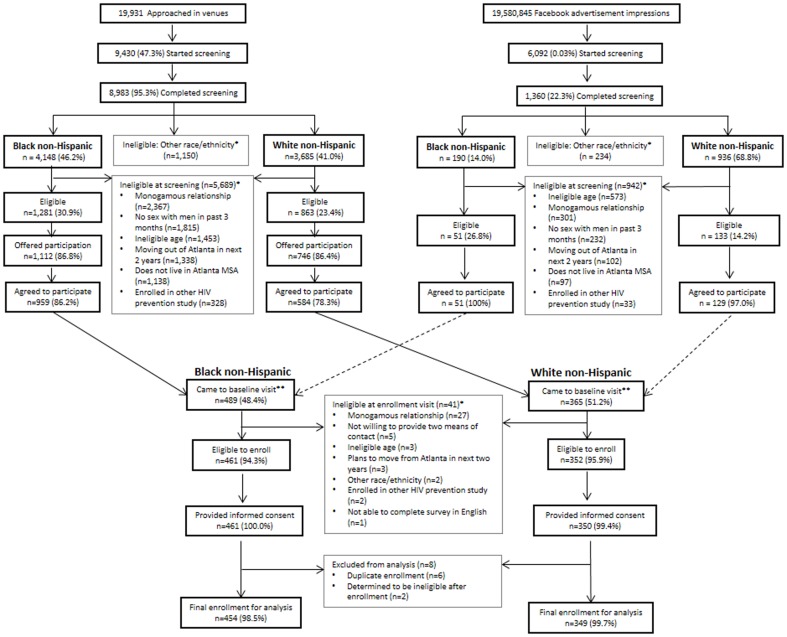
Enrollment scheme and results for recruitment of a black/white HIV/STI incidence cohort, Atlanta, 2010–2012. Men recruited through Facebook were screened for eligibility criteria online; men who met eligibility criteria in their online screening were invited to attend an in-person baseline (enrollment) visit. *Participant may have more than once reason for ineligibility. **The race of 3 participants was adjusted once they attended their baseline visit due to incorrect screening races being recorded.

Description of the InvolveMENt recruitment, screening and baseline enrollment outcomes are presented separately in Figure 1 for black and white MSM. Screening for InvolveMENt occurred from July 2010 through December 2012 at 94 individual venues and was comprised of 605 sampling events. Of 19,931 men approached at venues, 8,983 (45.1%) were screened, and 2,144 (23.9%) were eligible on initial screening (Figure 1). Of 6,092 men who clicked on the Facebook advertisement, 1,360 (22%) were screened, and 184 (13.5%) were eligible on initial screening. White men were more likely to be recruited by Facebook than were black men; however, there were no significant differences in education, socioeconomic status and confirmed HIV prevalence by recruitment method (data not shown in table). The most common reasons for ineligibility were being in a monogamous relationship with a man, not engaging in sex with a man in the past 3 months, and being either too old or too young. A total of 1,010 black MSM and 713 white MSM were offered participation in InvolveMENt and provided contact information. Approximately equal proportions of black and white MSM came to the baseline visit and consented to the study, resulting in 454 black MSM and 349 white MSM being enrolled and contributing to this analysis.

### Demographics and Behaviors

Black and white MSM significantly differed on all demographic characteristics except for recent incarceration (Table 1). Compared to white MSM, black MSM were younger, less likely to report being homosexual/gay, less likely to have a college degree, more likely to have been living in poverty, less likely to have been employed, less likely to have had health insurance and more likely to have been homeless. Black and white MSM did not significantly differ in reported arrest in the past 12 months (12.4% vs. 8.6%, p = 0.09). More than a quarter of black MSM reported current incomes below the federal poverty level, less than half had health insurance, and about 15% reported being homeless in the previous 12 months. Examining individual-level sex and drug use behaviors, black MSM reported significantly fewer male sex partners, fewer male unprotected anal intercourse partners, and lower levels of marijuana and other non-injection drug use in the previous 12 months, compared to white MSM. No significant differences were observed in the likelihood of reporting female sex partners, and white MSM were significantly more likely to report injection drug use, though this behavior was uncommon for both racial groups. No differences were observed in lifetime HIV testing, with more than 90% of MSM having ever tested for HIV, but black MSM were significantly less likely than white MSM to have had an HIV test in the previous 12 months.

**Table 1:** Demographic and behavioral characteristics of 803 black and white non-Hispanic MSM at enrollment in the *InvolveMENt* study.

	Black MSM (n = 454)		White MSM (n = 349)		
	*%*	*(total)*	*%*	*(total)*	p-value *
**Age** *(years)*		*(n = 454)*		*(n = 349)*	0.01
18–19	6.0	(27)	4.6	(16)	
20–24	34.4	(156)	26.1	(91)	
25–29	30.2	(137)	30.1	(105)	
30–39	27.5	(125)	34.7	(121)	
40+	2.0	(9)	4.6	(16)	
**Sexual identity**		*(n = 450)*		*(n = 349)*	<.0001
Homosexual/gay	77.8	(350)	93.1	(325)	
Bisexual	18.9	(85)	5.2	(18)	
Heterosexual/straight	0.2	(1)	0.6	(2)	
Other	3.1	(14)	1.1	(4)	
**Education**		*(n = 451)*		*(n = 348)*	<.0001
College, post-graduate, or professional school	29.9	(135)	54.0	(188)	
Some college, associate's degree, and/or technical school	44.6	(201)	35.6	(124)	
High school or GED	22.0	(99)	9.8	(34)	
Less than high school	3.5	(16)	0.6	(2)	
**Poverty, currently**	26.4	(97/367)	12.8	(41/321)	<.0001
**Employed, currently**	71.0	(318/448)	80.2	(280/349)	0.003
**Health Insurance, currently**	48.9	(215/440)	72.9	(253/347)	<.0001
**Homeless, current**	3.8	(17/449)	0.6	(2/348)	0.004
**Homeless, previous 12 months**	14.9	(67/451)	6.9	(24/347)	0.0005
**Arrested, previous 12 months**	12.4	(56/453)	8.6	(30/349)	0.09
**Recruitment venue type**		*(n = 454)*		*(n = 349)*	<.0001
Real-world venue	92.5	(420)	78.2	(273)	
Facebook	7.5	(34)	21.8	(76)	
Aggregate sexual behavior, previous 12 mo					
**Male sex partners** *median ([Q1, Q3], n)*					
** Total partners**	5	([3], [10], 451)	7	([4], [15], 346)	<.0001
** Unprotected anal intercourse partners**	1	([0, 3], 447)	2	([1], [3], 344)	0.003
**Any female sex partners** *% (total)*	5.7	(26/454)	5.4	(19/349)	0.86
Drug use, previous 12 months, self-report					
** Marijuana**	28.8	(130/451)	42.7	(147/344)	<.0001
** Other non-injection (non-poppers)** **	16.8	(72/429)	39.8	(136/342)	<.0001
** Injection**	0.7	(3/453)	3.5	(12/348)	0.006
HIV-testing history					
Lifetime	92.3	(417/452)	94.8	(331/349)	0.14
Lifetime (excl. HIV positive aware)	89.2	(289/324)	94.2	(293/311)	0.02
Previous 12 months (excl. HIV positive aware)	66.3	(214/323)	73.6	(229/311)	0.04

** Options for other non-injection drugs included: Crystal meth, crack, cocaine, downers, painkillers, hallucinogens, ecstasy, special K, GHB.

### Biomedical Measures

Results for biomedical measures are presented in Table 2. Compared to white MSM, black MSM had significantly higher prevalence of most STIs, being 3–4 times more likely to have urethral or rectal GC, rectal CT (95% CI: 1.4, 10.6), or a positive syphilis RPR result.

**Table 2:** Biomedical measures on 803 black and white non-Hispanic MSM at enrollment in the *InvolveMENt* study.

	Black MSM (n = 454)		White MSM (n = 349)				
	*%*	*(total)*	*%*	*(total)*	p-value *	*PR*	*[95% CI]*
**Circumcision status**, self-report	87.4	(368/421)	91.8	(301/328)	0.06	0.95	(0.91, 1.0)
Prevalent STI							
** Chlamydia, urethral**	2.7	(12/453)	2.9	(10/349)	0.85	0.92	(0.40, 2.12)
** Gonorrhea, urethral**	2.7	(12/453)	0.0	(0/349)	0.002	∞	(–)
** Chlamydia, rectal**	15.4	(33/214)	4.0	(4/100)	0.004	3.86	(1.40, 10.59)
** Gonorrhea, rectal**	10.8	(23/214)	3.0	(3/100)	0.03	3.58	(1.10, 11.65)
** Syphilis**							
RPR-positive	22.8	(103/452)	5.6	(20/348)	<.0001	3.97	(2.51, 6.27)
New (current/active) infection	1.6	(7/452)	0.9	(3/348)	0.53	1.80	(0.48, 11.51)
HIV infection							
HIV-positive	43.4	(197/454)	13.2	(46/349)	<.0001	3.29	(2.47, 4.40)
HIV-negative	56.6	(257/454)	86.8	(303/349)			
HIV clinical features							
** Engagement in care**					0.09		
Aware, in-care	53.3	(109/197)	73.9	(34/46)			
Aware, not in-care	6.1	(12/197)	6.5	(3/46)			
Aware, care unknown	4.1	(8/197)	2.2	(1/46)			
Unaware	34.5	(68/197)	17.4	(8/46)			
** CD4-count** *median ([Q1, Q3], n)*	490	([298, 642], 187)	577	([466, 692], 46)	0.007		
** log_10_ Viral Load** *median ([Q1, Q3], n)*	3.2	([1.4, 4.6], 198)	3.3	([1.4, 4.8], 46)	0.92		
Positive urine drug screening result	30.6	(139/454)	24.4	(85/349)	0.051	1.26	(1.00, 1.58)

HIV prevalence among black MSM was 43%, compared to 13% among white MSM (PR [95% CI]: 3.3 [2.5, 4.4]). There were 5 black MSM and 1 white MSM with primary HIV infection at enrollment. Among HIV-positive MSM, black MSM reported less awareness of and were therefore less likely to be in care for their infection than white MSM, although the overall patterns in care engagement were not statistically different. The median CD4 count of HIV-positive black MSM was significantly lower than that of white MSM and no significant differences were found in HIV viral load (Table 2).

When stratified by age, there was a striking increase in the age-specific prevalence of HIV between 18–19 year old and 20–24 year old black MSM (Table 3). Differences in the prevalence of HIV between black and white MSM were significant in all age groups except 18–19 years, and ≥40 years.

**Table 3:** Age-specific HIV prevalence among on 803 black and white non-Hispanic MSM at enrollment in the *InvolveMENt* study.

	Black MSM (n = 454)		White MSM (n = 349)				
	*%*	*(total)*	*%*	*(total)*	p-value *	*PR*	*[95% CI]*
**Age** *(years)*							
18–19	7.4	(2/27)	6.3	(1/16)	0.99	1.19	(0.11, 32.13)
20–24	34.0	(53/156)	5.5	(5/91)	<.0001	6.18	(2.57, 14.90)
25–29	45.3	(62/137)	14.3	(15/105)	<.0001	3.17	(1.92, 5.24)
30–39	60.0	(75/125)	15.7	(19/121)	<.0001	3.82	(2.47, 5.91)
40+	55.6	(5/9)	37.5	(6/16)	0.43	1.48	(0.55, 4.06)

### Dyadic Measures

At the baseline visit participants reported on 2,913 partnerships, with key features presented in Table 4. Black MSM were significantly more likely to have same race partners than white MSM. Black and white MSM had different distributions of partner types, with black MSM reporting relatively fewer main partners, and relatively more one-time casual partners, although absolute differences were small. Age concordance in dyads was not significantly different by participant race by the global CCC measure; however, white MSM were more likely than black MSM to have partnerships with an age discrepancy of more than 5 years (47% white vs. 38% black, p<.0001). There were no differences in the extent to which black and white MSM met partners online or in recent cohabitation with a sex partner. Levels of pre-sexual discussion of HIV serostatus and of perceived serostatus concordance were both significantly lower among black MSM.

**Table 4:** Characteristics of 2,913 partnerships reported by 803 black and white non-Hispanic MSM at enrollment in the *InvolveMENt* study.

	Black MSM (n = 1,617)		White MSM (n = 1,296)		
	*%*	*(total)*	*%*	*(total)*	p-value *
**Racially/ethnically concordant**	80.5	(1,256/1,561)	73.7	(939/1,274)	<.0001
**Age concordant** *Lin*'*s CCC (S.E.)*	0.37	(0.02)	0.32	(0.03)	0.11
**Partner Type**					0.03
Main	14.1	(223/1,583)	15.8	(202/1,281)	
Casual, repeat	29.6	(469/1,583)	32.9	(421/1,281)	
Casual, one-time	56.3	(891/1,583)	51.4	(658/1,281)	
**Met online**	39.2	(617/1,573)	40.9	(523/1,278)	0.36
**Cohabitation, previous 6 months**	5.9	(93/1,567)	6.6	(84/1,276)	0.48
**Pre-sexual serodiscussion**	51.6	(771/1,495)	70.2	(865/1,233)	<.0001
**Pre-sexual perceived seroconcordant**	40.1	(602/1,502)	61.4	(756/1,232)	<.0001

### Place-Based Measures

**Figure 2:**
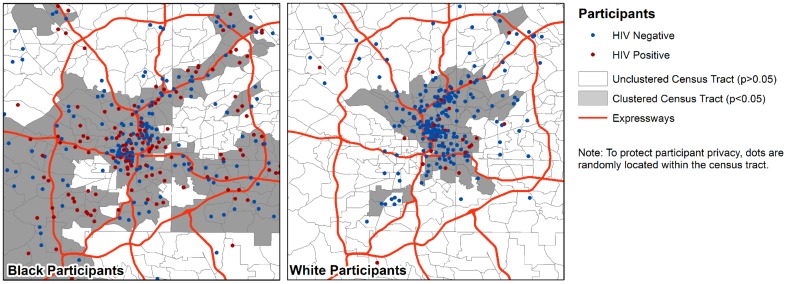
Distribution of residence of MSM enrolled in a cohort study of men who have sex with men in Atlanta, June 2010-December 2012.

Participants lived in a total of 350 of the 946 (37%) Atlanta census tracts at baseline, with the spatial distribution depicted in Figure 2. The statistically significant cluster of census tracts including black MSM was more diffuse than the statistically significant cluster of census tracts including white MSM and included more census tracts outside of Atlanta's urban core. The mean values of tract-level rates of poverty, high school graduation, and unemployment and median income all indicate that black MSM tended to live in more economically distressed census tracts than white MSM (Table 5). White MSM tended to live in census tracts that were home to more same-sex households and had higher ratios of men to women; they also lived in more densely populated tracts. Notably, there was no difference in the mean violent crime rate between racial groups, though white MSM had more spatial access to off-premises alcohol outlets. On average, black MSM lived in tracts with substantially higher percentages of other black residents. Black and white MSM lived in tracts with substantial prevalence of HIV diagnoses and although diagnosis rates in the tracts of white MSM were statistically higher, the absolute differences were small.

**Table 5:** Census tract characteristics of census tracts in which 797 black and white non-Hispanic MSM resided at enrollment in the *InvolveMENt* study.

	Black MSM (n = 448)		White MSM (n = 349)		
	*mean*	*(SD)*	*mean*	*(SD)*	*p-value*
Percent living in poverty	22.9	(13.3)	16.3	(12.8)	<.0001
Median annual household income	$42,902	($18,142)	$58,973	($22,742)	<.0001
Percent of adults with a high school degree/GED or less	41.7	(17.9)	26.5	(17.3)	<.0001
Percent of adults who are unemployed	12.1	(6.8)	7.2	(4.7)	<.0001
Alcohol outlet density, *per square mile*	6.8	(6.2)	9.1	(8.8)	<.0001
Violent crime rate, *per 1000 residents*	16.0	(19.8)	9.8	(10.0)	<.0007
Population density, *per square mile*	4,221	(3,465)	5,480	(4,547)	<.0001
Percent of residents who are non-Hispanic Black/African-American	62.2	(31.3)	27.7	(21.6)	<.0001
Percent of households containing a male same-sex couple	1.0	(1.1)	2.1	(1.7)	<.0001
Male:female sex ratio	0.98	(36.0)	1.18	(0.43)	<.0001
HIV diagnosis rate, *per 100,000 residents mean (SD, n)*	982.0	(759.0, 419)	1,185.1	(808.6, 289)	0.0007

A total of 350 unique census tracts were included in the analysis; there are 946 census tracts in the Atlanta MSA, which was the catchment area for the study. Because we calculated the mean of census tracts where the participants lived, the number of items of census tract data included in the average was equal to the number of participants for all calculations except for HIV diagnosis rate. Diagnosis rates are missing for 49 individuals who lived in census tracts not included in the data released from the state and for 40 individuals who lived in census tracts for which the numerator (number of persons living with an HIV infection diagnosis) was less than 5 and/or the denominator (number of people in the census tract in that population group) was <500.

## Discussion

Data from 803 black and white MSM confirmed the profound racial disparities in HIV and STI prevalence among MSM in Atlanta, and provided support for the utility of a multifactorial framework for understanding and eventually intervening to remediate disparities. This report of baseline data from the InvolveMENt cohort includes a description of the individual, dyadic, sexual network, and community-level factors that comprise this framework. This approach builds on recognition in the past decade that examining individual-level behavioral factors was likely to prove insufficient to explain or reduce black/white disparities in HIV infection [6]. Our results confirm that the differences that explain disparities in HIV between black and white MSM are likely to exist at the “higher-level” dyadic, sexual network, or neighborhood level rather than at the individual level. As others have reported and as we confirm, individual level differences exist between black and white MSM, but are not in the direction that would explain higher HIV prevalence and incidence among black MSM.

We used venue-time-space (VDT) sampling to identify men for screening and possible inclusion in the cohort [13]. This method, which has been widely used for cross-sectional behavioral surveillance surveys, has been infrequently used to recruit participants in longitudinal studies. Our aim was to use a sampling approach that avoided recruiting and enrolling clusters of men with correlated risk profiles, as might occur with recruitment focused in a limited sample of permissive venues. We tailored this approach in two ways. First, we used purposive selection of events to over-represent venues where black MSM congregate, based on our scientific aim to obtain a racially balanced cohort. A limited amount of purposive sampling is acceptable in VDT sampling [13]. Second, we expanded the traditional venue-time-space sampling approach by considering the Internet as an additional venue where we might locate the requisite number of MSM during our recruitment period. This modified recruitment strategy represents a logical repositioning of the traditional venue-time-space sampling approach, given the explosion of online social venues since the original MSM VTS protocol was developed. The Internet is no longer a marginal venue of sex-seeking MSM: a Pew survey indicated that 83% of Americans under the age of 29 had a social networking account, such as Facebook [25]. Using an Internet virtual “venue” also allowed us additional opportunities to recruit men aged 18-20, who are a critical group for HIV prevention according to epidemiologic trends [26]. These men are harder to reach in traditional venues, such as bars or dance clubs. Although there were significant differences in Facebook recruitment by race, our analyses revealed little potential for bias due to this. We found no significant differences in education, socioeconomic status and confirmed HIV prevalence by recruitment method. This relative lack of differences supports the notion that Facebook is a general social networking environment, rather than one that is systematically attended by higher-risk individuals. As a result, we feel that we developed a sample that met the scientific mandate for a racially balanced cohort and which reflects many important elements of the heterogeneous groups of MSM in Atlanta.

Our analysis of individual-level factors mainly confirmed previous observations that when differences in individual risk behaviors are observed between black and white MSM, they tend to favor higher HIV risk in white MSM. As we and others have reported, black MSM have fewer sex partners [27], fewer unprotected anal sex partners, less reported drug use, and equivalent levels of lifetime HIV testing [4], [6], [7], [28]. Black MSM reported less drug use in the surveys, but had higher findings of drugs metabolites in urine. This might be explained by differential misclassification of self-reported drug use by race; further analyses will be required to correlate these findings on an individual basis. Consistent with previous reports, we also observed that black MSM experience individual-level disparities in education, poverty, employment, health insurance, and current or recent homelessness.

For two individual factors, our results were more novel. Among our participants, black MSM were less likely to have tested for HIV in the past 12 months, and were equally likely to have been arrested in the past 12 months. With respect to testing, the preponderance of previous research has suggested that black MSM are equally likely or more likely to have been recently tested for HIV compared to white MSM. Our analysis excluded participants who were HIV-positive, but reported being unaware of their status. It is possible that some participants might have reported they were HIV-negative because of social desirability, or because they desired to participate in the prospective component of the study (i.e., they were not truly unaware of their infection). If this happened differentially among black MSM, the resulting misclassification would inflate the number of men in the black MSM group who would be less likely to have been tested in the prior year (because they knew themselves to be HIV-positive).

The other unexpected finding among our participants is that black MSM were not significantly more likely to report being arrested within the previous 12 months. Previous studies have provided mixed results with respect to this question. Some prior studies, including a large analysis of data from the National HIV Behavioral Surveillance System (NHBS) [29], have concluded that black MSM are more likely to experience criminal justice involvement than are white MSM [30]. A subsequent analysis of NHBS did not find significant differences in incarceration between black and white men who had been newly diagnosed with HIV [31]. It is possible that our study was underpowered to detect a true difference in history of recent arrest; the observed proportions of recent arrest are in the same direction, and with the same order of magnitude, of differences reported by NHBS [29]: in the 2003–2005 NHBS study in Atlanta, 7.2% of MSM reported recent arrest, and overall 10.6% of our respondents reported the same. The same item was used in both studies.

Our biological measures also largely confirmed previous findings. Our findings of a higher prevalence of urethral GC, rectal CT and GC, and serologic history of syphilis are all consistent with a long history of disproportionate impact of STIs among black Americans, best illustrated by surveillance data, and among black MSM, illustrated through many field investigations [32], [33]. Beyond racial disparities, it is worth noting that the absolute prevalence of undiagnosed rectal STIs was high in both black and white MSM. Further, our observation of the stark increase in HIV prevalence between 18–19 year and 20–24 year old black MSM, consistent with the reports of others [34] and with HIV incidence data [35], suggests that HIV prevention services for black MSM need to be made available to men much earlier, perhaps before young men reach the age of 18. We call on others, including public health agencies, to analyze and report age-stratified HIV prevalence data from MSM by race.

Rectal STI infections are often asymptomatic, and screening for rectal STIs is not standard of care in many clinical settings in Atlanta. Among HIV positive MSM, we did not observe a racial difference in engagement in care or in viral suppression, but we did observe a significantly lower baseline CD4 count among black MSM. The lack of difference in viral suppression is an important observation because it again represents parity for an individual-level difference that, if racially different, might explain higher risk for HIV transmission among black MSM. Of note, we have previously examined differences in the cascade of HIV care between black and white MSM in the InvolveMENt study and proposed additional metrics to better capture differences in HIV transmission risk [36].

Our data on dyadic level factors point to substantive differences in the patterns of sexual partnering by race, but they also refute some earlier hypotheses about network-level risks. For example, there are important differences in the extent of racially concordant partnerships, such that black MSM are much more likely to report sexual partnerships with other black men – who, as a group, have a higher prevalence of HIV infection. However, we did not find evidence for greater disassortativity by age in the partnerships of black MSM; some have hypothesized that high disassortativity by age among black MSM might favor higher transmission of HIV from older (higher prevalence) to younger (lower prevalence) black MSM [6]. The significance of the different distribution of partner types for black and white MSM is not clear: white men reported a higher proportion of their sex partners were main partners, but main partners might be a predominant source of new HIV transmissions within US MSM [37], [38]. We also found evidence that the extent of pre-sexual discussion of HIV serostatus is lower among black MSM than white MSM; this has also been reported previously by our group among other cohorts of Internet-using MSM [39]. Further aspects of sexual network structure, including concurrency (having temporally overlapping partnerships) [40], [41] and transitivity (the extent to which one's sex partners have sex with each other), might also be important, and will be explored further among our participants.

The most striking conclusion of the results of place-based characteristics is their consistency, which reflect the well-established socio-economic differences between white and black US populations – and their relationship to health [42]. All twelve of the place-based measures were different for the census tracts of black and white MSM. For the most part, these place-based differences depict that our black MSM participants lived in census tracts with lower income and education, and higher poverty and unemployment. The finding that black MSM lived in census tracts with much higher proportions of black residents is a place-level finding that is correlated with the higher observed prevalence of racially concordant sexual partnerships. Whether the higher observed prevalence of racially concordant relationships is related to characteristics of census tract of residence or to other factors, such as racism and stigma [43], is unclear. White MSM tended to be more highly clustered in Midtown Atlanta, where the census tracts have very high HIV prevalence. White MSM were also more likely to live in census tracts with counts that were suppressed due to sparse diagnoses (2% black vs. 8% white MSM lived in tracts with suppressed data), and were more likely to live in tracts outside of the City of Atlanta, where census tract-level diagnoses were not available (5% black vs. 9% white MSM).

Our study has a number of important limitations. Although we used venue-time-space sampling to ensure a systematic and reproducible approach to recruitment, our participants are not representative of all MSM in Atlanta. Our external validity is further limited by our enrollment criteria, which excluded men in monogamous partnerships and men ≥40 years of age. Since the design of the study, the important role of main partnerships in HIV transmissions among MSM has become clearer [37], [38]. Our measures that were collected by self-report – especially those related to illegal behaviors, such as drug use – are subject to social desirability bias [44]. For the 50 men who were negative for HIV antibodies at baseline and who did not attend a subsequent follow-up visit, it is possible that their HIV status was misclassified as negative when they were in fact primary HIV infections. If so, the extent of this misclassification was likely small: only 5/238 screened HIV-negative black MSM and 1/286 screened HIV-negative white MSM who did return for a second study visit were subsequently determined to have been HIV infected at their baseline visit. Our data on rectal STIs are incomplete because we did not begin rectal swab collection until approximately one year into the baseline recruitment of the study.

In this analysis we aimed to illustrate how a multi-level framework, inspired by Bronfrenbrenner's theoretical framework, could be used to systematically investigate the reasons for black/white disparities in HIV infections among MSM. We also sought, through descriptive analyses of archetypal characteristics at each theoretical level, to illustrate the theoretical levels at which black/white disparities are most likely to be generated or perpetuated. We concluded, as did a recent meta-analysis of behaviors and other factors among black and white MSM [28], that the most consistent black/white differences in the directions that favor more HIV transmission among black men occurred at the level of place. Millett et al illustrated that the highest effect sizes for relationships between various types of factors and disparities in HIV prevalence were for structural factors [28].

A key distinction in our consideration of racial and ethnic disparities relates to the difference between factors that might produce racial and ethnic disparities, and factors that perpetuate those disparities. For example, we observed that black MSM were more likely to report having black sex partners. If there were not a prior difference in HIV prevalence, this dyadic-level trait would not favor producing a black/white disparity in HIV transmissions. However, in our current state of existing disparity, this trait does support the perpetuation of those disparities. Understanding the factors that produce disparities will likely be difficult with contemporary datasets like ours. Our data are likely better suited to understanding perpetuating factors and, hopefully, to identifying new avenues for intervention.

Although the data presented here provide support for the utility of a multi-level framework to understand disparities and the importance of non-individual level factors in perpetuating black/white disparities, these analyses are not sufficient to fully explain why disparities exist – or how to remediate them. Next steps for analyses of our baseline data will include undertaking multi-level modeling, which might provide an understanding of how place-level characteristics relate to individual-level and network-level characteristics. These relationships are likely complex and will involve other conceptual domains, such as the relationship of stigma and place. Our hypothesis is that place-level characteristics shape lower levels in the theoretical model in ways that favor higher system risk of HIV acquisition for MSM. For example, the proportion of black residents in the census tract illustrates that most daily interactions within the neighborhoods of residence of black participants will be with other people of color. In turn, we observe that our black participants are much more likely to report black sex partners and have a greater extent of racially concordant partnerships than do our white participants. We have illustrated with partial data from our baseline cohort how the aggregation of these factors greatly increases the probability of black MSM having an unprotected anal sex act with the potential to transmit HIV [36]. More analyses such as this, using hierarchical linear modeling and other methods to illustrate how factors across levels interact, will further develop our critical understanding of these data.

Black/white disparities in HIV epidemics among MSM have been puzzling to public health officials and researchers for decades [4]. Understanding those disparities will require thinking broadly and exploring complex relationships among individual behaviors, dyadic and network characteristics, and community factors. This is particularly challenging because the disparities are occurring in environments with prevalent structural inequalities, functional segregation by race, and stigma. However, given what we know about the biological and epidemiological realities of HIV epidemics among MSM globally [45], it is unlikely that we will succeed in addressing disparities in infection without developing a deeper understanding of these factors. HIV has also been inexorably tied to stigmatization, marginalization, and lack of access. Based on our data, our ultimate understanding of black/white disparities in HIV infection of MSM will likely reprise many of these themes.

## Supporting Information

Figure S1 Questionnaire used to collect self-reported data elements in the Involve[men]t Study.
(PDF)

Table S1 Data sources used for place-based measures in the Involve[men]t Study.
(DOCX)
