# Supplementary material for: Understanding Racial HIV/STI Disparities in Black and White Men Who Have Sex with Men: A Multilevel Approach
Source: PLoS One. 2014 Mar 7;9(3):e90514. doi: 10.1371/journal.pone.0090514 (PMC3946498; doi:10.1371/journal.pone.0090514)
Supplement: Figure S1 — Questionnaire used to collect self-reported data elements in the Involve[men]t Study. (PDF) [file pone.0090514.s001.pdf]

Design, recruitment, and baseline characteristics of a prospective cohort study to understand racial HIV/STI disparities among black and white men who have sex with men in Atlanta, Georgia

Sullivan PS, et al.

Online supplement 1: Baseline survey instrument

This supplement contains the survey instrument for the baseline visit of the *InvolveMENt study*. The survey may be broadly broken into two sections: participant-specific and sexual partnership and network questions. The latter section contains questions about partners in aggregate, as well as partner-by-partner dyadic questions for the most recent 5 partners in the previous 6 months. Skip patterns are indicated where appropriate and the document also includes a number of data dictionary elements, such as variable names and coded values.

Please note that the correct pagination is indicated by the page numbers at the bottom center of each page. Additionally, the question numbers are non-sequential and should be ignored.

Correspondence regarding questionnaire content should be addressed to Patrick Sullivan (pssulli@emory.edu) or Eli Rosenberg (esrose2@emory.edu).

==
involveMENt - Baseline survey – **Participant-specific questions**
==

Contents

Demographic information

- Education and employment
- Income
- Race and sexual orientation
- Living situation
- Healthcare
- Arrest and incarceration

Substances

- Alcohol use and abuse (CAGE)
- Drug use and types
- Drug abuse screening test
- Substance use treatment

Circumcision

Harm reduction scales

Condoms

- Attitudes (Kalichman et al), and venue attendance
- Usage beliefs scale
- Norms (Peterson et al)
- Errors scale (Grimley et al)
- Condom skills for insertive sex scale

HIV

- Knowledge
- Testing
  - Testing history & results
  - Details of last test
- Care
  - Usual sources for HIV care
  - HIV treatment history

Perceived neighborhood scale (physical, social, services)

Perceptions of local stigma and local community (belongingness)

Resiliency

Racism and Life Experience

Depression

- BDI
- CESD

Sexual abuse (childhood and adulthood)

==
involveMENt - Baseline survey - **sexual partnership and network questions**
==

Contents

Partner metadata

- Partner genders, numbers, exchange sex
- Male partner metadata screen 2 (internet and sex acts)
- Male partner metadata screen 3 (UAI)

- Partner name list
- Partner calendar
- Concurrency clarification questions – if calendar indicates temporal ambiguities

Partner-specific section – *repeated for each recent sex partner named, up to 5*

Beginning of partnership

- Partner demographics
- Partnership description
- Geography
- Disclosure of status
- Serosorting intent

Partnership timing

- Date of first sex
- Date of last sex
- Ongoing relationship

Ongoing partnership questions

- Transgender partner anatomy
- Sex frequency in p6m
- Sexual agreements
- Sexual activity outside of this relationship
- Group sex
- STD diagnosis and treatment in p6m

Last sex

- Sexual activities
- Circumstances (location, drugs, HIV status)
- Last sex – HIV status knowledge
- HIV status knowledge source
- Strategic positioning

Post partner-specific wrap up

- Relationships among partners (transitivity)
- PrEP Usage
- Final screen

involve[men]t - Baseline survey

Staff login

InvolveMENt Staff:
Please log the participant in for his BASELINE visit by putting his full 7-digit study ID in the boxes below, including the dashes.

1. Study ID: (Required)

2. Repeat Study ID: (Required)

Key

Variable code names for SAS

Skip Patterns (#=question #)

Other skip pattern info

Hidden Question (participant does not see)

# = Question # for sections 2, 3

Format Name

Introduction

We're glad you're going to complete our survey today.

Helpful tips:

- Questions marked with are required questions that you must answer to move forward.
- This is a forward-only survey. Proceed to the next page by clicking the "Next Page" button. Please don't use the back button on your browser as this may cause problems with your survey.

Your privacy is important to us! All information you provide today in this survey will be held confidentially.

Education and Employment

5. What is the highest level in school that you completed? **educ** educ.

1 College, post graduate, or professional school
2 Some college, Associate's degree, and/or Technical school
3 High school or GED
4 Some high school
5 Less than high school
6 Never attended school
9 Don't Know

6. Have you had a paid job before? **paid_work_before** Binary_prefernot.

1 Yes — If yes, go to #7, #9, #10
0 No — If no, go to #8, #10
7 Prefer not to answer — If prefer, #7, #10

7. How would you describe you current work situation? (Check all that apply) — shown if Question #6= 1 or 7

Binary_prefernot.

- ☐ Full time paid job (>30 hours/week) — ***paid_fulltime***
- ☐ Part time paid job (<30 hours/week) — ***paid_parttime***
- ☐ Home duties/child care — ***paid_home***
- ☐ Full time student — ***paid_fullstu***
- ☐ Part time student — ***paid_partstu***
- ☐ Voluntary/charitable work — ***paid_vol***
- ☐ Have a job, but not at work due to extended illness, family leave, furlough or strike — ***paid_ill***
- ☐ Disabled — ***paid_disable***
- ☐ Unemployed for less than one year — ***paid_nojob_less12m***
- ☐ Unemployed for more than one year — ***paid_nojob_more12m***

Check = 1
Unchecked = 0

8. How would you describe you current work situation? (Check all that apply) — show #8 if paid_work_before=no, prefer not to answer

Binary_prefernot.

- ☐ Home duties/child care — nopay_home
- ☐ Full time student — nopay_fullstu
- ☐ Part time student — nopay_partstu
- ☐ Voluntary/charitable work — nopay_vol
- ☐ Disabled — nopay_disable
- ☐ Unemployed for less than one year — nopay_nojob_less1yr
- ☐ Unemployed for more than one year — nopay_nojob_more1yr

Check = 1
Unchecked = 0

For Q 7 and 8: Output will be checked="1" (yes) and unchecked="0" (no). Binary variable.

9. What kind of work do you do at your main job? ***work_type***
If you do not currently have paid work, what kind of work did you do at your last main job?

10. Do you make money from the underground economy (e.g., scams, numbers, drug dealing, prostitution)?

Binary_prefernot. ***under_econ***

1 Yes Skip to #11
0 No

shown if under_econ (#10) = 1

11. Briefly describe:

under_econ_desc

QUESTION INACTIVE

Page 4 of 218

Income

13. What was your household income last year from all sources before taxes?

(monthly / yearly)

00 0 to $417 (monthly) / 0 to $4,999 (yearly)
01 $418 to $833 (monthly) / $5,000 to $9,999 (yearly)
02 $834 to $1250 (monthly) / $10,000 to $14,999 (yearly)
03 $1251 to $1667 (monthly) / $15,000 to $19,999 (yearly)
04 $1668 to $2500 (monthly) / $20,000 to $29,999 (yearly)
05 $2501 to $3333 (monthly) / $30,000 to $39,999 (yearly)
06 $3334 to $4167 (monthly) / $40,000 to $49,999 (yearly)
07 $4168 to $6250 (monthly) / $50,000 to $74,999 (yearly)
08 $6251 or more (monthly) / $75,000 or more (yearly)
99 Don't know

Income

income.

14. Including yourself, how many people depended on this income? (must be at least 1)

dependent

Race and Sexual Orientation

Please tell us a little bit about yourself.

15. Do you think of yourself as: orient. orient

1 Heterosexual or "Straight"
2 Homosexual, Gay
3 Bisexual
4 Other ______ orient_other

16. There are different ways of referring to guys we know who have sex with men. We want to respect your preferences. During this survey, how would you like us to refer to men who have sex with other men? Please pick one of these choices:

callem

- Gay men
- Gay/bi men
- Same gender loving men
- Two-spirited men
- Queer men

Living Situation

17. Do you rent, own or stay at the place you rest at night? living living.

1 Rent
2 Own
3 Stay for nightly or monthly rate
4 Stay for free
5 Don't know

18. How long have you lived or stayed at the place you rest at night? living_duration living_duration

1 Less than one year
2 1-3 years
3 4-6 years
4 7-10 years
5 More than 10 years

19. What is the zip code for the place you stay at night? zipcode

20. In the past 12 months, have you been homeless at any time? By homeless, I mean you were living on the street, in a shelter, a Single Room Occupancy hotel (SRO), temporarily staying with friends or relatives, or living in a car? homeless Binary_prefernot.

1 Yes
0 No
9 Don't know

21. Are you currently homeless? homeless_now Binary_prefernot.

1 Yes
0 No
9 Don't know

Healthcare

22. Do you currently have health insurance? (This includes Medicare or Medicaid)

1 Yes — if yes, then #23
0 No — if no, then #24
9 Don't Know

insurance

Binary_prefernot.

23. What kind of health insurance or coverage do you currently have?

1 Private health insurance or HMO
2 Medicaid
3 Medicare
4 TRICARE (CHAMPUS)
5 Veterans Administration Coverage
6 Some other insurance, please specify: ______
7 Don't know

insurance_type.

insurance_type

insurance_other

24. Do you know where to go to be seen by a doctor, nurse or healthcare provider if you are sick?

4 Always
3 Most of the time
2 Sometimes
1 Not often
0 Never
7 Don't know

healthwhere

health_where.

25. Where do you primarily receive healthcare?

1 Healthcare provider office
2 Community clinic or health center
3 Hospital
4 Prison clinic
5 Commercial storefront clinic (ex. CVS, RiteAid, Walgreen's clinic)
6 Community-based organization
7 School clinic
8 Worksite health clinic
10 Local health department
9 Other ______

healthprimary

health_primary.

healthprimary_other

26. In the past 12 months, approximately how many times have you been treated for an illness or a condition by a healthcare provider?

0 0 times
1 1-4 times
2 5-9 times
3 10 times or more
7 Don't Know

times_treated

times_treated.

27. How often do you have enough money to pay for your prescription drugs?

money4meds

money4meds.

4 Always
3 Most often
2 Sometimes
1 Not often
0 Never
7 I don't need to buy prescription drugs at this time

28. How often do you have enough money to pay for healthcare provider office visits?

money4health

money4health.

4 Always
3 Most often
2 Sometimes
1 Not often
0 Never
7 Don't Know

Arrest and Incarceration

29. Have you ever been arrested?

1 Yes — If yes, then #30
0 No — If no, then #34

arrested_ever — Binary_prefernot.

30. In the past 12 months have you been arrested?

1 Yes — If yes, then #31, #32
0 No — If no, then #34

arrested_p12m — Binary_prefernot.

31. How many days did you spend in jail or prison the last time you were held?

1 Under 30 days
2 Over 30 Days
9 Don't know

arrested_jaildays — arrested_jaildays.

32. The last time you were held in jail or prison did you get a test for HIV?

1 Yes
0 No — If no, then #34
9 Don't know

arrested_jailtest — Binary_prefernot.

33. Did you get the results of that HIV test?

1 Yes
0 No
9 Don't know

arrested_jailresults — Binary_prefernot.

Alcohol use and abuse (CAGE)

34. During the past 12 months, have you had at least one drink of any alcoholic beverage such as beer, wine, a malt beverage or liquor?

drink_p12m — Binary_prefernot.

1 Yes — If yes, then #35
0 No — If no, then #36

35. Please check the one response to each item that best describes how you have felt and behaved over your whole life

	Yes	No
Have you ever felt you should cut down on your drinking? (drink_cut)	◯	◯
Have people annoyed you by criticizing your drinking? (drink_critic)	◯	◯
Have you ever felt bad or guilty about your drinking? (drink_guilt)	◯	◯
Have you ever had a drink first thing in the morning to steady your nerves or get rid of a hangover (eye-opener)? (drink_morning)	◯	◯
	1	0

Binary_prefernot.

Drug Use and Types 1

36. In the past 12 months, since [%%1742:month_11 %%], have you used any non-injection drugs, other than those prescribed for you?

If yes, then #37, #38

noninjection

Binary_prefernot.

1 Yes
0 No

37. In the past 12 months, since [%%1742:month_11 %%], how often did you use the following drugs?

drugfreq.		0 Didn't use	1 More than once a day	2 Once a day	3 More than once a week	4 Once a week	5 More than once a month	6 Once a month	7 Less than once a month
Crystal meth (tina, crank, or ice)	crystalmeth								
Crack cocaine	crack								
Powered cocaine that is smoked or snorted	cocaine								
Downers such as Valium, Ativan, or Xanax	downers								
Painkillers such as Oxycontin, Vicodin, or Percocet	painkillers								
Hallucinogens such as LSD or mushrooms	hallucinogen								
X or Ectasy	ectasy								
Special K (ketamine)	specialk								
GHB	ghb								
Heroin that is smoked or snorted	heroin								
Marijuana	maryjane								
Poppers (amyl nitrate)	poppers								

0 1 2 3 4 5 6 7

38. In the past 12 months, since [%%1742:month_11 %%], have you used any other non-injection drugs not listed above?

If yes, then #39, 40

noninject_other

Binary_prefernot.

1 Yes
0 No

39. Specify the non-injection drug you used, other than those listed above:

noninject_specify

Drug Use and Types 2

40. How often did you use [%%1958:Specify the non %%] ?

noninject_often

noninject_often.

1 More than once a day
2 Once a day
3 More than once a week
4 Once a week
5 More than once a month
6 Once a month
7 Less than once a month
8 Refused to answer
9

41. In the past 12 months, since [%%1742:month_11 %%], have you used Viagra, Levitra or Cialis?

viagra_p12m

Binary_prefernot.

1 Yes
0 No

42. In the past 12 months, since [%%1742:month_11 %%], have you used any drugs that you injected with a needle, other than those prescribed for you?

inject

Binary_prefernot.

1 Yes
0 No

http://www.empathia.com/providers/library/DrugAbuseScreeningTest.pdf

Drug abuse screening

43. Please check the one response to each item that best describes how you have felt over the past 12 months (since [%%1742:month_11 %%])

		Yes	No
Have you used drugs other than those required for medical reasons?	drug_noprescript		
Have you abused prescription drugs?	drug_abuse_prescript		
Do you abuse more than one drug at a time?	drug_multi		
Can you get through the week without using drugs?	drug_week		
Are you always able to stop using drugs when you want to?	drug_stop		
Have you had "blackouts" or "flashbacks" as a result of drug use?	drug_blackout		
Do you ever feel bad or guilty about your drug use?	drug_guilt		
Does your partner (or parents) ever complain about your involvement with drugs?	drug_complain		
Has drugs created problems between you and people close to you?	drug_prob		
Have you lost friends because of your use of drugs?	drug_friend		
Have you neglected people close to you because of your use of drugs?	drug_neglect		
Have you been in trouble at work because of your use of drugs?	drug_trouble		
Have you lost a job because of drug abuse?	drug_fired		
Have you gotten into fights when under the influence of drugs?	drug_fights		
Have you engaged in illegal activities in order to obtain drugs?	drug_legal		
Have you been arrested for possession of illegal drugs?	drug_arrest		
Have you ever experienced withdrawal symptoms (felt sick) when you stopped taking drugs	drug_withdrawal		
Have you had medical problems as a result of your drug use (e.g., memory loss, hepatitis, c	drug_medical		
Have you gone to anyone for help for a drug problem?	drug_help		
Have you been involved in a treatment program especially related to drug use?	drug_program		
	Binary_prefernot.	1	0

Substance use treatment

44. Have you ever participated in an alcohol or drug treatment program?

1 Yes If yes, then #45 drug_alcprogram Binary_prefernot.
0 No

45. Have you participated in an alcohol or drug treatment program in the past 12 months?

1 Yes drug_programp12m Binary_prefernot.
0 No

46. In the past 12 months, did you try to get into an alcohol or drug treatment program but were unable to?

1 Yes drug_noprogram_p12m Binary_prefernot.
0 No

Circumcision

47. Is your penis circumcised (cut) or uncircumcised (uncut)? circumcised circumcised.
Please select one choice.

1 Circumcised (cut) 0 Uncircumcised (uncut) 9 Don't know

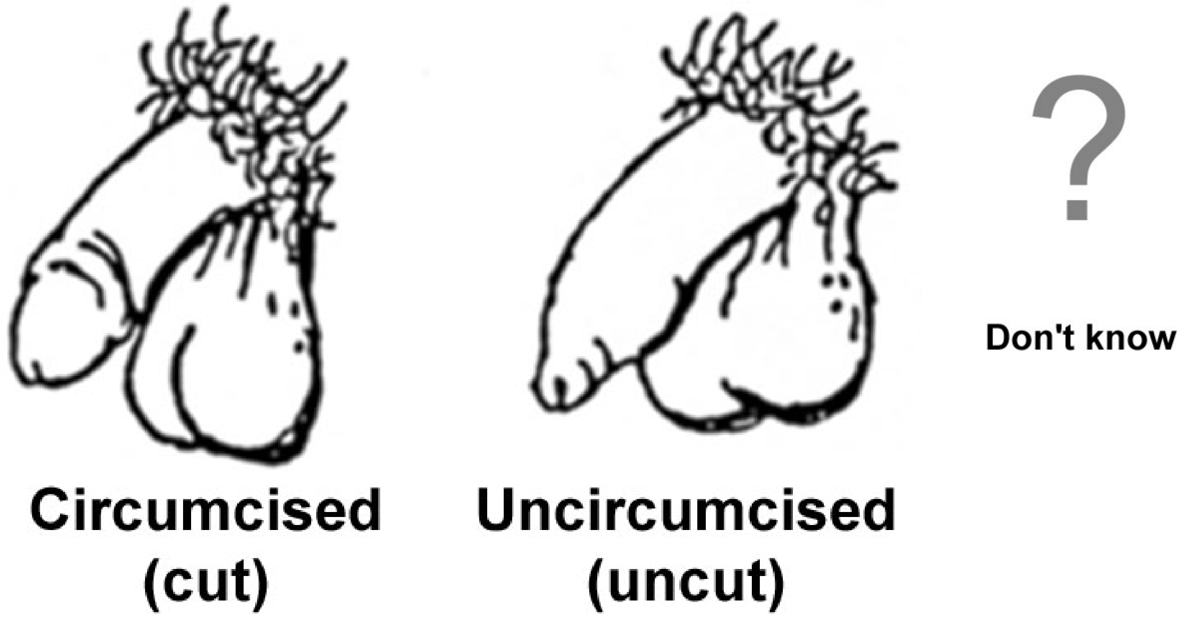

Harm reduction I

http://journals.lww.com/jaids/Fulltext/2001/12150/Influence_of_a_Partner_s_HIV_Serostatus,_Use_of.11.aspx
Suarez at al

60. If my partner tells me his HIV status is the same as mine:

HR_concordant_unsafe.

		Strongly disagree	Disagree	Somewhat disagree	Somewhat agree	Agree	Strongly agree
I am more likely to have unprotected sex with him	HR_concordant_unsafe	◯	◯	◯	◯	◯	◯
Then I worry less about HIV	HR_concordant_worry	◯	◯	◯	◯	◯	◯
		1	2	3	4	5	6

HR_concordant_worry.

Harm Reduction II

http://journals.lww.com/jaids/Fulltext/2001/12150/Influence_of_a_Partner_s_HIV_Serostatus,_Use_of.11.aspx
Suarez at al

48. You have met a man with whom you want to have sex and you don't know his HIV status .

Please rate how safe/unsafe you personally consider each of these sexual practices to be for you. Below are a number of sexual practices and a scale from 1 (completely safe) to 10 (completely risky). Please indicate the number that represents your view of how safe or risky each practice is for your health.

	Completely safe 1	2	3	4	5	6	7	8	9	Completely risky 10
You are the insertive partner during anal intercourse without a condom										
You are the insertive partner during condom-protected anal intercourse										
You are the receptive partner during anal intercourse without a condom										
You are the receptive partner during condom-protected anal intercourse										
You perform oral sex on a partner who cums in your mouth without a condom										
You perform oral sex on a partner WHO doesn't cum in your mouth										
Partner performs oral sex on you										
You engage in non-penetrative sex (mutual masturbation) until you and your partner cum										

1 2 3 4 5 6 7 8 9 10

HR_unk_UIAI
HR_unk_PIAI
HR_unk_URAI
HR_unk_PRAI
HR_unk_UOS
HR_unk_OS_nocum
HR_unk_ROS
HR_unk_mutualmast

harmreduction.

Harm Reduction III

http://journals.lww.com/jaids/Fulltext/2001/12150/Influence_of_a_Partner_s_HIV_Serostatus,_Use_of.11.aspx
Suarez at al

53. Earlier we asked you some questions about how safe some activities would be with a partner whose HIV status you did not know.

Now, please imagine that you have met a man with whom you want to have sex and he is HIV-negative.

Please rate how safe/unsafe you personally consider each of these sexual practices to be for you.

Below are a number of sexual practices and a scale from 1 (completely safe) to 10 (completely risky). Please indicate the number that represents your view of how safe or risky each practice is for your health.

	Completely safe 1	2	3	4	5	6	7	8	9	Completely risky 10
You are the insertive partner during anal intercourse without a condom	○	○	○	○	○	○	○	○	○	○
You are the insertive partner during condom-protected anal intercourse	○	○	○	○	○	○	○	○	○	○
You are the receptive partner during anal intercourse without a condom	○	○	○	○	○	○	○	○	○	○
You are the receptive partner during condom-protected anal intercourse	○	○	○	○	○	○	○	○	○	○
You perform oral sex on a partner who cums in your mouth without a condom	○	○	○	○	○	○	○	○	○	○
You perform oral sex on a partner who doesn't cum in your mouth	○	○	○	○	○	○	○	○	○	○
Partner performs oral sex on you	○	○	○	○	○	○	○	○	○	○
You engage in non-penetrative sex (mutual masturbation) until you and your partner cum	○	○	○	○	○	○	○	○	○	○
	1	2	3	4	5	6	7	8	9	10

HR_neg_UIAI
HR_neg_PIAI
HR_neg_URAI
HR_neg_PRAI
HR_neg_UOS
HR_neg_OS_nocum
HR_neg_ROS
HR_neg_mutualmast

harmreduction.

Harm Reduction IV

http://journals.lww.com/jaids/Fulltext/2001/12150/Influence_of_a_Partner_s_HIV_Serostatus,_Use_of.11.aspx
Suarez at al

58. Earlier we asked you some questions about how safe some sexual activities would be with partners who were HIV-negative or whose HIV status you did not know.

Now the question changes a litte.

Now, please imagine that you have met someone with whom you want to have sex and is HIV-positive, but taking antiretroviral medications that reduced his viral load to an undetectable level.

Please rate how safe/unsafe you personally consider each of these sexual practices to be for you.

Below are a number of sexual practices and a scale from 1 (completely safe) to 10 (completely risky). Please indicate the number that represents your view of how safe or risky each practice is for your health.

	Completely safe 1	2	3	4	5	6	7	8	9	Completely risky 10
You are the insertive partner during anal intercourse without a condom	◯	◯	◯	◯	◯	◯	◯	◯	◯	◯
You are the insertive partner during condom-protected anal intercourse	◯	◯	◯	◯	◯	◯	◯	◯	◯	◯
You are the receptive partner during anal intercourse without a condom	◯	◯	◯	◯	◯	◯	◯	◯	◯	◯
You are the receptive partner during condom-protected anal intercourse	◯	◯	◯	◯	◯	◯	◯	◯	◯	◯
You perform oral sex on a partner who cums in your mouth without a condom	◯	◯	◯	◯	◯	◯	◯	◯	◯	◯
You perform oral sex on a partner who doesn't cum in your mouth	◯	◯	◯	◯	◯	◯	◯	◯	◯	◯
Partner performs oral sex on you	◯	◯	◯	◯	◯	◯	◯	◯	◯	◯
You engage in non-penetrative sex (mutual masturbation) until you and your partner cum	◯	◯	◯	◯	◯	◯	◯	◯	◯	◯
	1	2	3	4	5	6	7	8	9	10

HR_pos_arv_UIAI
HR_pos_arv_PIAI
HR_pos_arv_URAI
HR_pos_arv_PRAI
HR_pos_arv_UOS
HR_pos_arv_OS_nocum
HR_pos_arv_ROS
HR_pos_arv_mutualmast

harmreduction.

Harm Reduction V

http://journals.lww.com/jaids/Fulltext/2001/12150/Influence_of_a_Partner_s_HIV_Serostatus,_Use_of.11.aspx
Suarez at al

61. Earlier we asked some questions about how safe you think some sexual activities are with different kinds of partners.

We have one more scenario to ask about.

This time, please imagine that you have met a man with whom you want to have sex and he is HIV-positive and not taking antiretroviral medications.

Please rate how safe/unsafe you personally consider each of these sexual practices to be for you.

Below are a number of sexual practices and a scale from 1 (completely safe) to 10 (completely risky). Please indicate the number that represents your view of how safe or risky each practice is for your health.

	Completely safe 1	2	3	4	5	6	7	8	9	Completely risky 10
You are the insertive partner during anal intercourse without a condom										
You are the insertive partner during condom-protected anal intercourse										
You are the receptive partner during anal intercourse without a condom										
You are the receptive partner during condom-protected anal intercourse										
You perform oral sex on a partner who cums in your mouth without a condom										
You perform oral sex on a partner who doesn't cum in your mouth										
Partner performs oral sex on you										
You engage in non-penetrative sex (mutual masturbation) until you and your partner cum										
	1	2	3	4	5	6	7	8	9	10

HR_pos_noarv_UIAI
HR_pos_noarv_PIAI
HR_pos_noarv_URAI
HR_pos_noarv_PRAI
HR_pos_noarv_UOS
HR_pos_noarv_OS_nocum
HR_pos_noarv_ROS
HR_pos_noarv_mutualmast

harmreduction.

Kalichman SC, Kelly JA, Rompa D. Continued high-risk sex among HIV seropositive gay and bisexual men seeking HIV prevention services. Health Psychol.1997; 16(4): 369-373.

Condom Attitudes, and venue attendance

49. The following statements ask much you agree or disagree with some ideas about condoms.

	<center>Strongly disagree<center>	<center> Moderately disagree<center>	<center>Slightly disagree<center>	<center>Neutral< center>	<center>Slightly agree<center>	<center> Moderately agree<center>	<center>Strongly agree<center>
The use of condoms can make sex more stimulating							
Condoms can be pleasurable							
Condoms go against my values or religious beliefs							
	1	2	3	4	5	6	7

condom_att_stimulate
condom_att_pleasure
condom_att_religious

Condom_Attitudes.

50. Which of these websites have you visited in the last month?
(select all that apply)

- MySpace
- Facebook
- Craigslist
- Adam4Adam
- Manhunt
- D-list
- FindFred
- Friendster
- Black Gay Chat / BGCLive.com
- OKCupid

onlinevenue_myspace
onlinevenue_facebook
onlinevenue_craigslist
onlinevenue_adam4adam
onlinevenue_manhunt
onlinevenue_dlist
onlinevenue_findfred
onlinevenue_friendster
onlinevenue_BGC
onlinevenue_OKC

Binary_Prefernot

Check = 1
Unchecked = 0

51. In the last month, in Atlanta, have you visited ...

	No	Yes
... **bars/restaurants** such as Joe's on Juniper, Marys, Gilberts, Django, Blake's, BJ Roosters, Burkharts, or Las Margaritas?		
... **gyms** such as Gravity, Urban Body Fitness, LA Fitness, or the Colony Square Athletic Club?		
... **clubs** such as Club Primal, The Jungle, Club Boi?		
... **social gatherings** such as Atlanta Executive Network, YouthPride, or private parties?		
... **outdoor locations** such as Piedmont Park or 10th & Piedmont?		
... **bath houses** such as Manifest, Club Eros, or Flex?		
	0	1

realworldvenue_bar
realworldvenue_gym
realworldvenue_club
realworldvenue_social
realworldvenue_outdoor
realworldvenue_bath

Binary_Prefernot

Condom usage beliefs

50. The following statements ask how much you approve or disapprove of some ideas about condom use.

	Strongly disapprove	Disapprove	Neutral	Approve	Strongly approve
Abstaining from sexual intercourse if condoms are not used by a new partner	○	○	○	○	○
Insisting on condom use even if your new partner does not want to use a condom	○	○	○	○	○
Not using a condom during sexual intercourse with a new partner	○	○	○	○	○
	1	2	3	4	5

condom_use_abstain
condom_use_insist
condom_use_nocondom

Condom_Use.

Condom norms (Peterson et al)

51. Please choose one answer for each statement.

	None of my friends	Less than half of my friends	About half of my friends	More than half of my friends	All of my friends
How many of your [%%3194:CALLEMmerged %%] friends always use condoms when having anal sex with a new partner?	○	○	○	○	○
How many of your [%%3194:CALLEMmerged %%] friends think you should avoid unsafe sex?	○	○	○	○	○
How many of the [%%3194:CALLEMmerged %%] men you know only engage in safe sex practices?	○	○	○	○	○
How may of your [%%3194:CALLEMmerged %%] friends think you should always have safe sex?	○	○	○	○	○
	1	2	3	4	5

norms_condom
norms_avoid
norms_onlysafe
norms_yousafe

Norms.

52. How important do your [%%3194:CALLEMmerged %%] friends think it is to use a condom when having anal sex with a new partner?

1 Not important at all
2 Slightly important
3 Moderately important
4 Very important
5 Extremely important

normal_PAI_new

Norms_PAI.

Condom Errors

Grimley DM, Annang L, Houser S, Chen H. Prevalence of condom use errors among STD clinic patients. AM J Health Behav. 2005; 29(4): 324-330.

54. Have you used a condom in the last 6 months?

condomp6m

Binary_prefernot.

1 Yes if yes, then #55, #56
0 No

55. In the last 6 months, when you used a condom, did you:

		Yes	No	Don't Know
Squeeze air from the tip of condom before putting it on?	condom_err_air	◯	◯	◯
Experience a condom breaking?	condom_err_break	◯	◯	◯
Hold the base of the condom during withdrawal?	condom_err_base	◯	◯	◯
Leave space at the tip of condom?	condom_err_space	◯	◯	◯
Completely unroll the condom before putting it on?	condom_err_unroll	◯	◯	◯
Start having sex, then put the condom on during intercourse?	condom_err_during	◯	◯	◯
Put condom on inside out, then flip it over to use?	condom_err_flip	◯	◯	◯
Re-use a condom?	condom_err_reuse	◯	◯	◯
		1	0	9

Binary_prefernot.

Condom Skills for Insertive Sex

a.k.a.: if #54 (condom6mo)=1(yes), then continue to #56

Complete this section only if: "CONLAST" matches: '1'

56. In the last 6 months, have you had anal sex as the insertive partner (top) and used a condom?

condom_err_analp6m

Binary_prefernot.

1 Yes if yes, then #55, #56
0 No

57. During the last six months when you used a condom for anal sex as a "top" did any of the following occur, even once?

Grimley DM, Annang L, Houser S, Chen H. Prevalence of condom use errors among STD clinic patients. AM J Health Behav. 2005; 29(4): 324-330.	Always	Very Often	Sometimes	Rarely	Never
Did you let a condom you were using touch sharp jewelry, fingernails or teeth?	○	○	○	○	○
Did you put a condom on your penis with the wrong side up and then have to flip it over before you could use it?	○	○	○	○	○
Did you completely unroll the condom before putting it on your penis?	○	○	○	○	○
Did you hold the tip of the condom to leave a space, then roll it down to the base of your penis?	○	○	○	○	○
Did you use a dry condom? (i.e. the condom was non-lubricated and you did not add any lubricant like KY jelly)	○	○	○	○	○
Did you add lubricant to the condom?	○	○	○	○	○
Did you use an oil-based lubricant such as Vaseline, baby oil, massage oil?	○	○	○	○	○
Did you have any problems with losing an erection while putting on a condom?	○	○	○	○	○
Did you have any problems with losing your erection once the condom was on and sex had begun?	○	○	○	○	○
Did you start having sex without a condom and then pull out and put one on?	○	○	○	○	○
Did you start having sex with a condom on and then take it off before sex was over?	○	○	○	○	○
Did the condom you were using break during sex?	○	○	○	○	○
Did the condom you were using slip off during sex?	○	○	○	○	○
Did the condom you were using slip-off after sex, as you were pulling out?	○	○	○	○	○
Did you have a problem with the way a condom fit or felt on you? (for example, you felt it was too small or too large, it was the wrong shape, it caused irritation of your skin or you/your partner couldn't feel anything with it on)?	○	○	○	○	○
	5	4	3	2	1

con_IAI_sharp
con_IAI_flip
con_IAI_unroll
con_IAI_base
con_IAI_dry
con_IAI_lube
con_IAI_oil
con_IAI_edputon
con_IAI_edsex
con_IAI_during
con_IAI_remove
con_IAI_break
con_IAI_slip
con_IAI_pullout
con_IAI_fit

LikertFive.

HIV Knowledge

Carey, MP., Schroder, KE. Development and psychometric evaluation of the brief HIV knowledge questionnaire. AIDS Educ Prev. 2002; 14(2): 172-182

59. The next set of questions ask about your HIV knowledge. For each statement, please click "True", "False", or "I don't know." If you do not know, please do not guess; instead, please click the button: "I don't know."

	True	False	Don't Know
Coughing and sneezing DO NOT spread HIV.	◯	◯	◯
A person can get HIV by sharing a glass of water with someone who has HIV.	◯	◯	◯
Pulling out the penis before a man climaxes/cums keeps his partner from getting HIV during sex.	◯	◯	◯
A woman can get HIV if she has anal sex with a man.	◯	◯	◯
Showering, or washing one's genitals/private parts, after sex keeps a person from getting HIV.	◯	◯	◯
All pregnant women infected with HIV will have babies born with AIDS.	◯	◯	◯
People who have been infected with HIV quickly show serious signs of being infected.	◯	◯	◯
There is a vaccine that can stop adults from getting HIV.	◯	◯	◯
People are likely to get HIV by deep kissing (putting their tongue in their partner's mouth), if their partner has HIV.	◯	◯	◯
A woman cannot get HIV if she has sex during her period.	◯	◯	◯
There is a female condom that can help decrease a woman's chance of getting HIV.	◯	◯	◯
A natural skin condom works better against HIV than does a latex condom.	◯	◯	◯
A person will NOT get HIV if he is taking antibiotics.	◯	◯	◯
Having sex with more than one partner can increase a person's chance of becoming infected with HIV.	◯	◯	◯
Taking a test for HIV one week after having sex will tell a person if she or he has HIV.	◯	◯	◯
A person can get HIV by sitting in a hot tub or a swimming pool with a person who has HIV.	◯	◯	◯
A person can get HIV from oral sex.	◯	◯	◯
Using Vaseline or baby oil with condoms lowers the chance of getting HIV.	◯	◯	◯
	1	0	9

HIVedu_sneeze
HIVedu_water
HIVedu_pullout
HIVedu_ladyanal
HIVedu_douche
HIVedu_pregnant
HIVedu_quickill
HIVedu_vaccine
HIVedu_french
HIVedu_period
HIVedu_femalecon
HIVedu_skin
HIVedu_antibio
HIVedu_multiple
HIVedu_window
HIVedu_hottub
HIVedu_oral
HIVedu_babyoil

Binary_tfdk.

HIV Testing History and Results

62. Have you ever been tested for HIV? HIVtest_ever Binary_prefernot.

1 Yes If yes, then #63, #64, #65, #66
0 No

63. In what month and year did you have your most recent HIV test?

Month: HIVtest_month HIVtest_month.

January	=1
February	=2
March	=3
April	=4
May	=5
June	=6
July	=7
August	=8
September	=9
October	=10
November	=11
December	=12

64. Year: HIVtest_year

65. What was the result of your most recent HIV test? HIVtest_result HIVtest_result.

0 Negative
1 Positive If positive, then hide #67, #70, #71, #72, #73. If positive, then show #68, #69
2 Indeterminant/Inconclusive
3 Didn't get the results of my last HIV test

Details of Last HIV Test

Complete this section only if: "EVERTEST" matches: '1'

If "ever_tested" = 1(yes), then show this page

66. When you got tested in [%%1901: %%] [%%1902: %%], where did you get tested?

1 Private doctor's office (including HMO)
2 Community health center/public health clinic
3 HIV counseling and testing site
4 HIV/AIDS street outreach program/Mobile Unit
5 Hospital (inpatient)
6 Emergency room
7 Sexually transmitted disease clinic
8 Drug treatment program
9 Correctional facility (jail or prison)
10 Blood bank/Plasma center
11 Military
12 At home
13 Other ______

HIVtest_where

HIVtest_where.

HIVtest_where_other

67. How likely is it that you'll get tested for HIV in the next 12 months?

1 Very likely
2 Somewhat likely
3 Somewhat unlikely
4 Very unlikely

HIV_next12mo

HIV_next12mo.

Usual Sources of Care for HIV

Complete this section only if: "HRESULT" matches: '1'

68. In the past 12 months, is there one place in particular, like a doctor's office or clinic, where you usually go for most of your HIV care, like CD4 tests, viral load tests, or prescriptions for HIV medicines?

1 Yes
0 No If no, then #69

HIVcare_oneplace

Binary_prefernot.

69. What are the reasons you don't have a usual source of care for HIV? [Check all that apply]

- ☐ Couldn't afford a usual source of care — nocare_cost
- ☐ Didn't know where to find regular HIV care — nocare_findcare
- ☐ Couldn't get a regular appointment anywhere — nocare_appt
- ☐ No HIV doctors in my area — nocare_nodoc
- ☐ Didn't think it was necessary — nocare_neces
- ☐ Thought it was necessary, but never tried to get one — nocare_notry
- ☐ Didn't know where to find a regular doctor who speaks the same language as me — nocare_language
- ☐ Have just recently been diagnosed — nocare_newdiag
- ☐ Other (specify) ____________ — nocare_other

Binary_prefernot.

Check = 1
Unchecked = 0

Binary_prefernot.

HIV Treatment History

Complete this section only if: "HRESULT" matches: '1'

70. Have you ever taken any antiretroviral medicines to treat your HIV? These medicines are also known as ART, HAART, or the AIDS cocktail.

1 Yes — If yes, then #72
0 No — if no, then #71

arv_ever — Binary_prefernot.

71. What are the reasons you have never taken any antiretroviral medicines?

Option	Variable
☐ Doctor advised to delay treatment	noarv_ever_doctor
☐ Recently into medical care/haven't had time	noarv_ever_notime
☐ CD4 count and/or viral load are good	noarv_ever_cd4_good
☐ Feel good, don't need them	noarv_ever_feelgood
☐ Worried about side effects	noarv_ever_sidefx
☐ Drinking or using drugs	noarv_ever_drugs
☐ Didn't want to think about being HIV positive	noarv_ever_denial
☐ No money	noarv_ever_cost
☐ No insurance	noarv_ever_noins
☐ Worried about ability to adhere/often forget	noarv_ever_adhere
☐ Living on the street	noarv_ever_homeless
☐ Taking alternative/complementary medicines	noarv_ever_altmed
☐ Don't know	noarv_ever_dontknow
☐ Other (specify) ______	noarv_ever_other

Binary_prefernot.

Check = 1
Unchecked = 0

72. Are you currently taking any antiretroviral medicines to treat your HIV?

1 Yes
0 No — if no, then #73

arv_current — Binary_prefernot.

73. What are the reasons you aren't currently taking any antiretroviral medicines? [Check all that apply]

Option	Variable
☐ Doctor advised to delay treatment	noarv_now_doctor
☐ Recently into medical care/haven't had time	noarv_now_notime
☐ CD4 count and/or viral load are good	noarv_now_cd4_good
☐ Feel good, don't need them	noarv_now_feelgood
☐ Worried about side effects	noarv_now_sidefx
☐ Drinking or using drugs	noarv_now_drugs
☐ Didn't want to think about being HIV positive	noarv_now_denial
☐ No money	noarv_now_cost
☐ No insurance	noarv_now_noins
☐ Worried about ability to adhere/often forget	noarv_now_adhere
☐ Living on the street	noarv_now_homeless
☐ Taking alternative/complementary medicines	noarv_now_altmed
☐ Other (specify) ______	noarv_now_other

Binary_prefernot.

Check = 1
Unchecked = 0

Perceived Neighborhood

74. Please rate your neighborhood's physical environment as it is now with the regard to the following:

		Excellent	Above average	Average	Below average	Poor
Affordable and comfortable housing	hood_housing	○	○	○	○	○
Public parks or open spaces	hood_parks	○	○	○	○	○
Noise	hood_noise	○	○	○	○	○
Crowdedness	hood_crowd	○	○	○	○	○
Air quality	hood_air	○	○	○	○	○
		1	2	3	4	5

hood.

75. Please rate your neighborhood's social environment as it is now with regard to the following:

		Excellent	Above average	Average	Below average	Poor
Friendliness/helpfulness of neighbors	hood_friendly	○	○	○	○	○
Residents' attachment to the neighborhood	hood_attach	○	○	○	○	○
Self-esteem and morale of the residents	hood_morale	○	○	○	○	○
Personal safety	hood_safety	○	○	○	○	○
Opportunity to participate in local government	hood_citizenship	○	○	○	○	○
Ability to have access to city councilman/alderman	hood_govaccess	○	○	○	○	○
		1	2	3	4	5

hood.

76. Please rate your neighborhoods' service environment as it is now with regard to the following:

		Excellent	Above average	Average	Below average	Poor
Quality of schools	hood_school	○	○	○	○	○
Policing	hood_police	○	○	○	○	○
Access to hospital/medical care	hood_healthcare	○	○	○	○	○
Shopping	hood_shopping	○	○	○	○	○
Lighting	hood_light	○	○	○	○	○
Garbage pickup/snow removal	hood_garbage	○	○	○	○	○
		1	2	3	4	5

hood.

Perceptions of Local Stigma & Local Community Perceptions

77. Please answer each of the following items by checking the box that best fits your response.

	Strongly disagree	Disagree	Neutral	Agree	Strongly agree	Not Applicable
Most people in my city/town believe that a [%%3194:CALLEMmerged %%] man is just as trustworthy as the average heterosexual citizen	◯	◯	◯	◯	◯	◯
Most employers in my city/town will hire a [%%3194:CALLEMmerged %%] man if he is qualified for the job	◯	◯	◯	◯	◯	◯
Most people in my city/town feel that homosexuality is a sign of personal failure	◯	◯	◯	◯	◯	◯
Most people in my city/town would not hire a [%%3194:CALLEMmerged %%] man to take care of their children	◯	◯	◯	◯	◯	◯
Most people in my city/town think less of a person who is [%%3194:CALLEMmerged %%]	◯	◯	◯	◯	◯	◯
Most people in my city/town would treat a [%%3194:CALLEMmerged %%] man just as they would treat anyone	◯	◯	◯	◯	◯	◯
Most people in my city/town will willingly accept a [%%3194:CALLEMmerged %%] man as a close friend	◯	◯	◯	◯	◯	◯
	1	2	3	4	5	8

78. Please answer each of the following items by checking the box that best fits your response.

	Strongly disagree	Disagree	Neutral	Agree	Strongly agree	Not Applicable
I feel that I am a member of my city/town gay community	◯	◯	◯	◯	◯	◯
I plan to stay in my city/town for a long time	◯	◯	◯	◯	◯	◯
I have many [%%3194:CALLEMmerged %%] male friends in my city/town	◯	◯	◯	◯	◯	◯
I have many lesbian/bisexual women in my city/town	◯	◯	◯	◯	◯	◯
I wish that I could live someplace with a stronger gay/bisexual community than the place I live	◯	◯	◯	◯	◯	◯
I regularly attend gay events and meetings in my city/town	◯	◯	◯	◯	◯	◯
My town/city is a bad place for me to live as a [%%3194:CALLEMmerged %%] man	◯	◯	◯	◯	◯	◯
I feel at home in my city/towns' gay community	◯	◯	◯	◯	◯	◯
As a [%%3194:CALLEMmerged %%] man, I enjoy living in my city/town	◯	◯	◯	◯	◯	◯
	1	2	3	4	5	8

Question 77 -Variable Names

comm.

comm_trust
comm_hire
comm_failure
comm_child
comm_opinion
comm_treat
comm_friend

Question 78 - Var Names

comm.

comm_member
comm_stay
comm_gayfriends
comm_lesbian
comm_bettercom
comm_events
comm_badplace
comm_athome
comm_enjoy

Resiliency

http://www.resiliencescale.com/en/rstest/rstest_14_en.html

79. Please check the box indicating how much you disagree or agree with each statement

		Strongly disagree	Disagree	Neutral	Agree	Strongly agree	Not Applicable
I usually manage one way or another	res_manage	◯	◯	◯	◯	◯	◯
I feel proud that I have accomplished things in my life	res_proud	◯	◯	◯	◯	◯	◯
I usually take things in stride	res_stride	◯	◯	◯	◯	◯	◯
I am friends with myself	res_selflove	◯	◯	◯	◯	◯	◯
I am determined	res_determined	◯	◯	◯	◯	◯	◯
I keep interested in things	res_interest	◯	◯	◯	◯	◯	◯
My belief in myself gets me through hard times	res_persevere	◯	◯	◯	◯	◯	◯
My life has meaning	res_meaning	◯	◯	◯	◯	◯	◯
When I am in a difficult situation, I can usually find my way out of it	res_getout	◯	◯	◯	◯	◯	◯
I have enough energy to do what I have to do	res_energy	◯	◯	◯	◯	◯	◯
		1	2	3	4	5	NA

resiliency.

Racism and Life Experience I

80. Please answer each of the following items by checking the box that best fits your response.

In the past 12 months.....

	Never	Rarely	Sometimes	Fairly Often	Very Often
How often have your civil rights been violated (i.e., job or housing discrimination due to racism, racial discrimination, or racial prejudice?	◯	◯	◯	◯	◯
How often have others said or acted as if you are over-sensitive or paranoid about racism?	◯	◯	◯	◯	◯
How often have you witnessed prejudice or discrimination directed at someone else because of their race/ethnic group?	◯	◯	◯	◯	◯
How often have you heard about someone else's experiences of racially-motivated discrimination or prejudice?	◯	◯	◯	◯	◯
How often have others reacted to you as if they were afraid or intimidated of you because of your race/ethnic group?	◯	◯	◯	◯	◯
	1	2	3	4	5

racism.

racism_rights
racism_paranoid
racism_witness
racism_another
racism_fear

Racism and Life Experience II

81. Please answer each of the following items by checking the box that best fits your response.

In the past 12 months.....

	Never	Rarely	Sometimes	Fairly Often	Very Often	Prefer Not to Answer
How often have you been observed or followed while in public places because of your race/ ethnic group?	◯	◯	◯	◯	◯	◯
How often have you been treated as if you were “stupid” or “talked down to” because of your race/ethnic group?	◯	◯	◯	◯	◯	◯
How often have your ideas or opinions been minimized, ignored, or devalued because of your race/ethnic group?	◯	◯	◯	◯	◯	◯
How often have you heard (or been told) a racially offensive or insensitive comment or joke?	◯	◯	◯	◯	◯	◯
How often have you been mistaken for someone who serves others (i.e., janitor, bellboy, maid) because of your race/ethnic group?	◯	◯	◯	◯	◯	◯
How often have you been mistaken for someone else of your same race (who may not look like you at all)?	◯	◯	◯	◯	◯	◯
	1	2	3	4	5	7

racism_prefer.

racism_stared
racism_stupid
racism_ignored
racism_insensitive
racism_service
racism_mistaken

BDI-PC

This next section contains groups of statements. Please read each group of statements carefully. Then pick out the one statement in each group which best describes the way you have been feeling the PAST TWO WEEKS, INCLUDING TODAY. Select the box beside the statement you picked. If several statements in the group seem to apply equally well, select each one.

Be sure to read all the statements in each group before making your choice.

82. Describe the way you have been feeling for the past two weeks, including today.

- ☐ I do not feel sad
- ☐ I feel sad
- ☐ I am sad all the time and I can't snap out of it
- ☐ I am so sad or unhappy that I can't stand it

Check = 1
Unchecked = 0

bdi_sad0
bdi_sad1
bdi_sad2
bdi_sad3

Binary_prefernot.

83. Describe the way you have been feeling for the past two weeks, including today.

- ☐ I am not particularly discouraged about the future
- ☐ I feel discouraged about the future
- ☐ I feel I have nothing to look forward to
- ☐ I feel the future is hopeless and that things cannot improve

Check = 1
Unchecked = 0

bdi_future0
bdi_future1
bdi_future2
bdi_future3

Binary_prefernot.

84. Describe the way you have been feeling for the past two weeks, including today.

- ☐ I do not feel like a failure
- ☐ I feel I have failed more than the average person
- ☐ As I look back on my life, all I can see are a lot of failures
- ☐ I feel I am a complete failure as a person

Check=1
Unchecked=0

bdi_failure0
bdi_failure1
bdi_failure2
bdi_failure3

Binary_prefernot.

85. Describe the way you have been feeling for the past two weeks, including today.

- ☐ I get as much satisfaction out of things as I used to
- ☐ I don't enjoy things the way I used to
- ☐ I don't get satisfaction out of things anymore
- ☐ I am dissatisfied or bored with everything

bdi_satisfaction0
bdi_satisfaction1
bdi_satisfaction2
bdi_satisfaction3

Binary_prefernot.

Check = 1
Unchecked = 0

86. Describe the way you have been feeling for the past two weeks, including today.

- ☐ I don't feel disappointed in myself
- ☐ I am disappointed in myself
- ☐ I am disgusted in myself
- ☐ I hate myself

Check = 1
Unchecked = 0

bdi_disappointed0
bdi_disappointed1
bdi_disappointed2
bdi_disappointed3

Binary_prefernot.

87. Describe the way you have been feeling for the past two weeks, including today.

- ☐ I don't feel I am any worse than anybody else
- ☐ I am critical of myself for my weaknesses or mistakes
- ☐ I blame myself all of the time for my faults
- ☐ I blame myself for everything bad that happens

bdi_faults0
bdi_faults1
bdi_faults2
bdi_faults3

Binary_prefernot.

Check = 1
Unchecked = 0

88. Describe the way you have been feeling for the past two weeks, including today.

- ☐ I don't have any thoughts of killing myself
- ☐ I have thoughts of killing myself, but I would not carry them out
- ☐ I would like to kill myself
- ☐ I would kill myself if I had the chance

Check = 1
Unchecked = 0

bdi_suicide0
bdi_suicide1
bdi_suicide2
bdi_suicide3

Binary_prefernot.

CESD

89. Please choose the answer that best fits how you have felt and behaved during the past week

		Rarely or none of the time </br>(<1 day)	Some or little of the time </br>(1-2 days)	Occasionally or a moderate amount of the time </br>(3-4 days)	Most or all of the time </br>(5-7 days)
I was bothered by things that don't usually bother me	cesd_bother				
I had trouble keeping my mind on what I was doing	cesd_concentrate				
I felt depressed	cesd_depressed				
I felt everything I did was an effort	cesd_effort				
I felt hopeful about the future	cesd_hopeful				
I felt fearful	cesd_fearful				
My sleep was restless	cesd_badsleep				
I was happy	cesd_happy				
I felt lonely	cesd_lonely				
I could not get "going"	cesd_getgoing				
		0	1	2	3

CESD.

Sexual Abuse

90. The following questions deal with topics that are often difficult to talk or think about. They are questions about physical and sexual abuse that you may have experienced early in your life. When the question says, “child,” it means when you were age 16 or younger.

		Yes	No
As a child, were you ever beaten, physically attacked, or physically abused?	abuse_child_physabuse	◯	◯
As a child, were you ever sexually attacked, raped, or sexually abused?	abuse_child_sexabuse	◯	◯
As an adult, have you ever been beaten, physically attacked, or physically abused?	abuse_adult_physabuse	◯	◯
As an adult, have you ever been sexually attacked, raped, or sexually abused?	abuse_adult_sexabuse	◯	◯
		1	0

Binary_prefernot.

Open text field to make corrections

521. You are almost finished with our survey!

We understand that having a forward-only survey can be frustrating because it does not allow you to make corrections to previous responses.

If you would like to change something that you've told us, please describe the question(s) and what the response(s) should be, using the space below. survey_changes

==
involveMENt - Baseline survey - **sexual partnership and network questions**
==

<u>Contents</u> (repeated for convenience)

<u>Partner metadata</u>

- Partner genders, numbers, exchange sex
- Male partner metadata screen 2 (internet and sex acts)
- Male partner metadata screen 3 (UAI)

- Partner name list
- Partner calendar
- Concurrency clarification questions – if calendar indicates temporal ambiguities

<u>Partner-specific section</u> – *repeated for each recent sex partner named, up to 5*

<u>Beginning of partnership</u>

- Partner demographics
- Partnership description
- Geography
- Disclosure of status
- Serosorting intent

<u>Partnership timing</u>

- Date of first sex
- Date of last sex
- Ongoing relationship

<u>Ongoing partnership questions</u>

- Transgender partner anatomy
- Sex frequency in p6m
- Sexual agreements
- Sexual activity outside of this relationship
- Group sex
- STD diagnosis and treatment in p6m

<u>Last sex</u>

- Sexual activities
- Circumstances (location, drugs, HIV status)
- Last sex – HIV status knowledge
- HIV status knowledge source
- Strategic positioning

<u>Post partner-specific wrap up</u>

- Relationships among partners
- PrEP usage
- Final screen

==
Partner genders and numbers – p12m *(Baseline only)*
==

Thank you for all that you have told us so far. These next questions ask about people you've had sex with during the last 12 months, **since the beginning of [%%1742:month_11 %%]**.

Binary_prefernot.

93 During the last 12 months, did you have sex with a person who was*:*
(mark all that apply)

Check = 1
Unchecked = 0

( ) Male
( ) Female
( ) Transgender: male to female
( ) Transgender: female to male

sexp12m_m
sexp12m_f
sexp12m_mtf
sexp12m_ftm

if male, then #94, #95
if female, then #96, #97
if trans mtf=#98, #99
if trans ftm=#100, #101

If 'male':

94 During the last 12 months, with how many men did you have anal or oral sex?:

__________ male_howmanyp12m

Were any of these male sex partners an exchange partner -- that is a partner that you have sex with in exchange for money, drugs, food, or something else of value?

95
1 ( ) Yes
0 ( ) No
9 ( ) Don't know

male_exchangep12m

Binary_prefernot.

If 'female':

96 During the last 12 months, with how many women did you have vaginal or anal sex?:

__________ female_howmanyp12m

97 Were any of these female sex partners an exchange partner -- that is a partner that you have sex with in exchange for money, drugs, food, or something else of value?

1 ( ) Yes
0 ( ) No
9 ( ) Don't know

female_exchangep12m

Binary_prefernot.

If 'Transgender: male to female':

98 During the last 12 months, with how many male to female transgender individuals did you have sex?:

__________ mtf_howmanyp12m

99 Were any of these male to female transgender sex partners an exchange partner -- that is a partner that you have sex with in exchange for money, drugs, food, or something else of value?

1 ( ) Yes
0 ( ) No
9 ( ) Don't know

mtf_exchangep12m

Binary_prefernot.

If 'Transgender: female to male'

100 During the last 12 months, with how many female to male transgender individuals did you have sex?:

__________ ftm_howmanyp12m

101 Were any of these female to male transgender sex partners an exchange partner -- that is a partner that you have sex with in exchange for money, drugs, food, or something else of value?

1 ( ) Yes
0 ( ) No
9 ( ) Don't know

ftm_exchangep12m

Binary_prefernot.

[if > 0 male partners in p12m. Actually this is always true at Baseline]
==
Partner classification and numbers – male partners p12m *(Baseline only)*
==

102 Of the [%%45:%%] male partners you had anal or oral sex with in the last 12 months, how many were:

[ ] main partners? (someone that you feel committed to above all others -- this is someone you might call your boyfriend, significant other, life partner, or husband) male_mainp12m

[ ] casual partners? (someone that you do not feel committed to above all others) male_casualp12m

[continuous sum shows total]

Please ensure that the total number of main + casual partners equals [%%45:During the last %%]. Your current total equals [%%249:total msp %%]. if male_totalp12m=0, then #108 male_totalp12m

==
Male partner metadata screen 2 – p12m *(Baseline only)*
==

103. Of the [%%45:During the last %%] male partners you had anal or oral sex with in the last 12 months, how many did you meet on the Internet? male_internetp12m

____

The total number of sex partners met on the Internet cannot be more than the total number of male sex partners ([%%45:During the last %%]).

104 Of the [%%45:During the last %%] male partners you had sex with in the last 12 months, how many did you have anal sex with? male_AIp12m

if male_analp12m (#104)=0, then #108
if male_analp12m=>1, skip #107
if male_analp12m=1, #107

____

105 Of the [%%45:During the last %%] male partners you had sex with in the last 12 months, how many did you have oral sex with? male_OSp12m

____

The total number of anal sex partners cannot be more than the total number of male sex partners ([%%45:During the last %%]).

==
Male partner metadata screen 3 - p12m *(Baseline only)*
==
If >1 male AI partner:

106 Of your [%%49:Of the [%% %%] partners you had anal sex with in the last 12 months, how many did you have unprotected anal sex with? (This means that you or your partner did not use a condom at any point during sex, at least one time that you had anal sex)

males_UAIp12m

____

ERROR: The number of male partners you had unprotected anal sex with can't be more than the total number of anal sex partners ([%%49:Of the [%% %%]).

If 1 male AI partner:

107 In the last 12 months, did you have unprotected anal sex with your male anal sex partner? (This means that you or your partner did not use a condom at any point during sex, at least one time that you had anal sex)

1 ( ) Yes
0 ( ) No
9 ( ) Don't know

male_UAIp12m

Binary_prefernot.

==
Transition from p12m to p6m metadata recall *(Baseline only)*
==

Thank you for telling us about your partners in the last 12 months.

Next, we'd like to ask you about your partners in the last 6 months (since the beginning of XXX).

Some of these questions will be very similar to earlier ones, but please keep in mind that they are now referring to partners you had since the beginning of [XXX]

Click Next Page to continue.

==
Partner genders and numbers – p6m
==

108 During the <u>last 6 months</u>, did you have sex with a person who was:
(mark all that apply)

( ) Male
( ) Female
( ) Transgender: male to female
( ) Transgender: female to male

Check = 1
Unchecked = 0

sexp6m_m
sexp6m_f
sexp6m_mtf
sexp6m_ftm

if male=#109, #110
if female=#111, #112
if trans mtf=#113, #114
if trans ftm=#115, 116

Binary_prefernot.

If 'male':

109 During the last 6 months, with how many men did you have anal or oral sex?:

__________ male_howmanyp6m if 0=#123

110 Was any of these male sex partners an exchange partner -- that is a partner that you have sex with in exchange for money, drugs, food, or something else of value?

1 ( ) Yes
0 ( ) No
9 ( ) Don't know

male_exchangep6m

Binary_prefernot.

[if "yes" selected then all <u>male</u> partner-specific exchange questions are suddenly visible (<u>set default</u> to they are off)]

If 'female':

111 During the last 6 months, with how many women did you have vaginal or anal sex?:

__________ female_howmanyp6m

112 Was any of these female sex partners an exchange partner -- that is a partner that you have sex with in exchange for money, drugs, food, or something else of value?

1 Yes
0 No
9 Don't know

female_exchangep6m

Binary_prefernot.

[if "yes" selected then all <u>female</u> partner-specific exchange questions are suddenly visible (<u>set default</u> to they are off)]

If 'Transgender: male to female':

113 During the last 6 months, with how many male to female transgender individuals did you have sex?:

__________ mtf_howmanyp6m

114 Was any of these [%%45:During the last %%] transgender: male to female sex partners an exchange partner -- that is a partner that you have sex with in exchange for money, drugs, food, or something else of value?

1 Yes
0 No
9 Don't know

mtf_exchangep6m

Binary_prefernot.

[if "yes" selected then all transgender: male to female partner-specific exchange questions are suddenly visible (<u>set default</u> to they are off)]

If ‘Transgender: female to male’

115 During the last 6 months, with how many female to male transgender individuals did you have sex?:

__________ ftm_howmanyp6m

116 Was any of these [%%45:During the last %%] transgender: female to male sex partners an exchange partner -- that is a partner that you have sex with in exchange for money, drugs, food, or something else of value?

1 Yes
0 No
9 Don't know

ftm_exchangep6m

Binary_prefernot.

[if “yes” selected then all transgender: female to male partner-specific exchange questions are suddenly visible (set default to they are off)]

[if > 0 male partners in p6m]

==

Partner classification and numbers – non-transgender male partners p6m

==

117 Of the [%%45:%%] male partners you had anal or oral sex with in the last 6 months, how many were:

[ ] main partners? (someone that you feel committed to above all others -- this is someone you might call your boyfriend, significant other, life partner, or husband) male_mainp6m

[ ] casual partners? (someone that you do not feel committed to above all others.) male_casualp6m

[continuous sum shows total]

Please ensure that the total number of main + casual partners equals [%%45:During the last %%]. Your current total equals [%%249:total msp %%]. male_totalp6m

==
Partner metadata screen 2 – p6m
==

118 Of the [%%45:During the last %%] male partners you had anal or oral sex with in the last 6 months, how many did you meet on the Internet?

male_internetp6m

____

The total number of sex partners met on the Internet cannot be more than the total number of male sex partners ([%%45:During the last %%]).

119 Of the [%%45:During the last %%] male partners you had sex with in the last 6 months, how many did you have anal sex with?

if #119=0, then #123
if #119=>1=#121, skip #122
if #119=1, then #122

male_AIp6m

____

120 Of the [%%45:During the last %%] male partners you had sex with in the last 6 months, how many did you have oral sex with?

male_OSp6m

____

The total number of anal sex partners cannot be more than the total number of male sex partners ([%%45:During the last %%]).

==
Partner metadata screen 3 – p6m
==

If >1 male AI partner:

121 Of your [%%49:Of the [%% %%] partners you had anal sex with in the last 6 months, how many did you have unprotected anal sex with? (This means that you or your partner did not use a condom at any point during sex, at least one time that you had anal sex).

males_UAIp6m

____

ERROR: The number of male partners you had unprotected anal sex with can't be more than the total number of anal sex partners ([%%49:Of the [%% %%]).

If 1 male AI partner:

122 In the last 6 months, did you have unprotected anal sex with your male anal sex partner? (This means that you or your partner did not use a condom at any point during sex, at least one time that you had anal sex)

( ) Yes
( ) No
( ) Don't know

male_UAIp6m

==
Intro partner list
==

This next section will be about some of your recent sex partners, regardless of gender.

In order to do that, we will ask you to provide nicknames for your recent sex partners. We will then use these nicknames to customize the questions so that they are specific to your partners.

It is important that you choose a nickname that will best help you remember the person. The names you provide are meant to help you only and should not reveal your partner’s full identity. We do not want to know who your partners actually are.

Some examples of nicknames that you might choose are: a partner’s first name, a nickname you call the partner by, the place where you both met or an online screen name.

===
Partner name list
===

Please give a nickname for each of your most recent sex partners over the last 6 months (since the beginning of [%%432:month_5 %%]).

Male, female, and transgender sex partners may be in this list:
For male partners, we mean people you had oral or anal sex with.
For female partners, we mean people you had vaginal, or anal sex with.

123 Space for 5 partners is provided, but you only need to fill in as many spaces as you need or can remember.

If you had more than 5 sex partners in the previous 6 months, we would like nicknames for the most recent 5.

Partner 1 (most recent) ________________
Partner 2 ________________
Partner 3 ________________
Partner 4 ________________
Partner 5 ________________

p1
p2
p3
p4
p5

name_count

==
Partner calendar
==

124 For each sex partner listed, indicate, each month you had sex with that partner

Calendar created as follows:

Each names partner has a row
Each of the last 6 months is provided in a column
Response is required in each row

	**Oct '09**	**Nov '09**	**Dec '09**	**Jan '10**	**Feb '10**	**Mar '10**
<Partner_name_1>	[ ]	[ ]	[ ]	[ ]	[ ]	[ ]
<Partner_name_2>	[ ]	[ ]	[ ]	[ ]	[ ]	[ ]
<Partner_name_3>	[ ]	[ ]	[ ]	[ ]	[ ]	[ ]
<Partner_name_4>	[ ]	[ ]	[ ]	[ ]	[ ]	[ ]
<Partner_name_5>	[ ]	[ ]	[ ]	[ ]	[ ]	[ ]

p1m5, p1m4, p1m3....p2m5, p2m4...etc.

If there are no ambiguous partnerships with one-month overlaps:
==
Proceed to partnership-specific questions
==

Thank you for telling us the months in which you had sex with your partners.

Click Next to continue

skip to “Intro. to partner-specific questions“

If there are ambiguous partnerships with one-month overlaps:
==
Proceed to concurrency clarification questions
==

Thank you for telling us the months in which you had sex with your partners.

The next page will ask some questions to help us better understand what you just told us about your sexual partnerships.

Click Next to continue

==
Concurrency clarification questions
==

For each ambiguous partnership that has a one-month overlap, this question is provided:

You indicated that you had sex with both *[partner X]* and *[partner Y]* in the month of *[Month Z].*

125 Which of these statements about *[Month Z]* is most correct?

concurrency_clarify

1 I last had sex with *[partner X]* before I had sex with *[partner Y]*
2 I last had sex with *[partner Y]* before I had sex with *[partner X]*
3 I was having sex with both *[partner X]* and *[partner Y]* during the same time period
9? Don't know

==
Intro. to partner-specific questions
==
On the next pages, we would like to ask you some questions about the sex partners for whom you gave nicknames.

For each partner you named, you will be asked similar questions. Some questions will be about the partner in general and some will be about your relationship with the partner.

The first questions will be about [%%403_O0:Space for 10&nb %%].

Click Next Page to begin.

===
Partner Demographics: #1
===

201 Is [%%454:cur_partner_nam %%] male, female, or transgender?
1 Male
2 Female
3 Transgender: male to female
4 Transgender: female to male

genderp1

202. What is [%%454:cur_partner_nam %%]'s current age?

(if you an unsure of the exact age, choose an age that you think is close)

agep1

if #202=don't know then #203

____

If "don't know"

203 Which of the following statements about [%%454:cur_partner_nam %%]'s age is most true?
1 He or she is more than 10 years <u>younger</u> than I am
2 He or she is 2-10 years <u>younger</u> than I am
3 He or she is within a year of my age
4 He or she is 2-10 years <u>older</u> than I am
5 He or she is more than 10 years <u>older</u> than I am
9 Don't know

agestatementp1

204 Is [%%454:cur_partner_nam %%] Hispanic?
1 Yes
0 No
9 Don't know

hispanicp1

205 What race is [%%454:cur_partner_nam %%]? (check one)
1 ( ) Asian/Pacific Islander
2 ( ) Black/African-American
3 ( ) White/Caucasian
4 ( ) Native American/Alaska Native
5 ( ) Mixed Race
6 ( ) Other ← racep1N5
9 ( ) Don't know

racep1

If "mixed race"

206 You indicated that [%%454:cur_partner_nam %%] is of mixed race. Which terms best describe [%%454:cur_partner_nam %%]?
1 Asian/Pacific Islander
2 Black/African American
3 White/Caucasian
4 American Indian/Alaskan Native
6 Other

asian_p1
black_p1
white_p1
native_p1
other_p1

207 Has [%%454:cur_partner_nam %%] had a paid job before?
1 Yes
2 No
9 Don't know

paidjob_p1

if #207=yes, then #208
if #207=no, then #209
if don't know, then #208

If "Yes"

208 How would you describe [%%454:cur_partner_nam %%]'s current work situation?

(Check all that apply)

1 ( ) Full time paid job (>30 hours/week) — work_fulljob_p1 — if fulltime then #210
2 ( ) Part time paid job (<30 hours/week) — work_part_p1 — if parttime then #210
3 ( ) Home duties/child care — work_home_p1
4 ( ) Full time student — work_fullstu_p1
5 ( ) Part time student — work_pstu_p1
6 ( ) Voluntary/charitable work — work_volunt_p1
7 ( ) Have a job, but not at work due to extended illness, family leave, furlough or strike — work_leave_p1 — if illness then #210
8 ( ) Disabled — work_disabled_p1 — if disabled then #210
9 ( ) Unemployed for less than one year — work_less1yr_p1 — if unemployed less 1yr then #210
10 ( ) Unemployed for more than one year — work_more1yr_p1 — if unemployed more 1yr then #210
99 ( ) Don't know — work_dontknow_p1

210 What kind of work does or did [%%454:cur_partner_nam %%] do on his/her last main job?

________________________________ mainjobp1

If "no"

209 How would you describe [%%454:cur_partner_nam %%]'s current work situation?

(Check all that apply)

3 Home duties/child care — nopay_home_p1
4 Full time student — nopay_fullstu_p1
5 Part time student — nopay_pstu_p1
6 Voluntary/charitable work — nopay_volunt_p1
7 Disabled — nopay_disabled_p1
8 Unemployed for less than one year — nopay_less1yr_p1
9 Unemployed for more than one yea — nopay_more1yr_p1
10 Don't know — nopay_dontknow_p1
99

211 In the last 6 mo[illegible]s (since the beginning of [%%432:month_5 %%]), has [%%454:cur_partner_nam %%] been arrested?

1 Yes — if yes=#212
0 No
9 Don't know

arrestP1

If 'yes':

212 How many days did [%%454:cur_partner_nam %%] spend in jail or prison the last time [%%454:cur_partner_nam %%] was held?

1 Under 30 days
0 Over 30 days
9 Don't know

daysjailP1

==
Partnership description: #1
==

Please tell us a little about **you and [%%454:cur_partner_nam %%]**

213 Did you have sex with [%%454:cur_partner_nam %%] once, or more than once during the last 6 months?

0 ( ) Once
1 ( ) More than once
REQUIRED

if once=#216
if more than once=#214

morethanonceP1

If 'more than once', then ask: (one-time partners automatically casual)

214 Is/was [%%454:cur_partner_nam %%] someone that you feel or felt committed to above all others (someone you might call your boyfriend, significant other, life partner, or husband)?

mainP1

0 ( ) Yes
1 ( ) No
9 ( ) Don't know
REQUIRED

if #95,#97,#99,#101=yes, then #215
if #95,#97,#99,#101=no or 9, then #216

If 'partner classification and numbers' section above reveals exchange partners, then ask:

215 72. Is/was [%%454:cur_partner_nam %%] an exchange partner (someone who you have sex with in exchange for money, drugs, food, or something else of value)?

0 ( ) Yes
1 ( ) No
9 ( ) Don't know
REQUIRED

exchangeP1

216 If you had to further describe the type of sex partner [%%454:cur_partner_nam %%] is/was, which of the following would you choose?

gorbachP1

Someone who …
1 ( ) … is your primary sexual partner
2 ( ) … you have sex with on a regular basis, <i>but who is not your main or primary partner</i>
3 ( ) … you have had sexual contact with more than once, <i>but not on a regular basis</i>, and who you <u>normally socialize with</u>
4 ( ) … you have had sexual contact with more than once, <i>but not on a regular basis</i>, and who you <u>don't socialize with</u>
5 ( ) … you had sexual contact with only 1 time, but could find again if necessary
6 ( ) … you had never met before you had had sexual contact and never plan to see again
7 ( ) … you gave sex to for money or other goods or someone who gave you sex for money or other goods

217 Please rate the strength of your relationship with [%%454:cur_partner_nam %%] on a scale from 1 (weak) to 10 (strong):

relstrengthp1

1 1 (very weak)
2 2
3 3
4 4
5 5
6 6
7 7
8 8
9 9
10 10 (very strong)

==
Geography: #1
==

218 Where did you first meet [%%454:cur_partner_nam %%]?
wheremeetP1 – P5

wheremeetP1

- ( ) Through friends 1
- ( ) School or work 2
- ( ) Circuit party or Rave 3
- ( ) At church 4
- ( ) Online 5
- ( ) Through a personal ad in a newspaper 6
- ( ) On a telephone chat line or dating line 7
- ( ) Bar/Club 8
- ( ) Cruising area 9
- ( ) On the street 10
- ( ) Adult bookstore 11
- ( ) Bath house 12
- ( ) Sex club 13
- ( ) Sex resort 14
- ( ) Private sex party 15
- ( ) Sports club or gym 16
- ( ) Vacation or cruise 17
- ( ) Social organization 18
- ( ) Other wheremeet_P118 20

219 *if "Internet" …*
Through which online service did you first meet [%%454:cur_partner_nam %%]?

onlineservice P1

- 1 ( ) MySpace
- 2 ( ) Facebook
- 3 ( ) CraigsList
- 4 ( ) Adam4Adam
- 5 ( ) Manhunt
- 6 ( ) D-list
- 7 ( ) FindFred
- 8 ( ) Friendster
- 9 ( ) Grindr
- 20 ( ) Other, *please specify:* ________ onlineservice P1N9

onlineserviceP1 – P5

if the partnership type was not "anonymous"(SG note: done in script):

220 Did you and [%%454:cur_partner_nam %%] live together at any point during the last 6 months (since the beginning of [%%432:month_5 %%])?

livetogetherp1

- 1 Yes
- 0 No

livetogetherP1 – P5

if #213=yes, then phrasing 1
if #213=no, then phrasing 2

==
Disclosure of status
==

221 *if had sex "more than once" above:*
Did you and [%%454:cur_partner_nam %%] share both of your HIV statuses before you first had sex?

if had sex "once" above:
Did you and [%%454:cur_partner_nam %%] share both of your HIV statuses before you had sex?

discussstatusfsP1

1 ( ) Yes
0 ( ) No
9 ( ) Don't know
discussstatusfsP1 – P5

if yes then #222

222 *If 'yes' to discussing status:*
What was [%%454:cur_partner_nam %%]'s status at that time?

statusfsP1

1 HIV-negative
0 HIV-positive
9 Don't know
statu[illegible]1 – P5

if pos or neg, then #223
if don't know, then #225

If participant and his partner discussed their HIV statuses and partner was positive or negative, this page is shown. Otherwise skipped to next page:

==
Serosorting intent
==

223 *if had sex "more than once" above:* statusimpP1

How important was the knowledge that [%%454:cur_partner_nam %%] was [partner's HIV status] in deciding to first have sex with [%%454:cur_partner_nam %%]?%%]?

if had sex "once" above:

How important was the knowledge that [%%454:cur_partner_nam %%] was [partner's HIV status] in deciding to have sex with [%%454:cur_partner_nam %%]?

1 Not important at all
2 Slightly important
3 Moderately important
4 Very important
5 Extremely important

statusimpP1 – P5

224 *Online involveMENt pilot only. Done for ONLY first partner: if answers the above question:*

We are trying to develop new questions to help understand the decisions that men make when choosing their sex partners.

Was this question clear to you? statisimpclearP1

1 Yes
0 No, *please explain and feel free to offer any suggestions:* ________________

statusimpclearP1

if #213=once, then #234
if #213=more than once, then #225

[If had sex "more than once", then the participant next completes the sections pertaining to dates of first and last sex].
[If had sex "once", then the participant is skipped to the "Ongoing relationship" section"].
==
Date of first sex: #1
==

76. When did you first have sex with [%%454:cur_partner_nam %%]?

You may enter just the month and year, but if you know the exact date, please enter that instead.

If you are unsure of when you first had sex, try to select a time that you think is close.

It is OK if you first had sex with [%%454:cur_partner_nam %%] longer than 6 months ago; we still would like to know when you first had sex.

firstsexmoP1

225 (drop-down box with months. "don't know the month" is provided as option) ***firstsexmoP1***
226 (drop-down box with years) "don't know the year" is provided as option) ***firstsexyrP1*** firstsexyrp1

OR

227 MM/DD/YYYY (calendar button) ***firstsexdtP1*** firstsexdtP1

[ERROR: The time you first had sex with [%%454:cur_partner_nam %%] cannot be in the future. Please correct the date.]

[ERROR: Please select either the month and year OR the date you first had sex with [%%454:cur_partner_nam %%]]

if don't know then #228,#229

Completed only for non-one-time partners

===

Date of first sex – unknown month or year: #1

===

228 *if year is selected as "don't know the year"* firstsexyearsP1

It's OK if you can't remember the exact year.

Can you remember about how many years ago you first had sex with [%%454:cur_partner_nam %%]?

1 Less than 1 year ago
2 1 – 2 years ago
3 2 – 5 years ago
4 5 – 10 years ago
5 more than 10 years ago

firstsexyearsP1 – P5
(REQ)

229 *if month is selected as "don't know the month" (but year is known)*

It's OK if you can't remember the exact month.

Think back to the time in [Year of first sex] when you first had sex with [%%454:cur_partner_nam %%].

Perhaps you had sex around a special time of the year such as your birthday, or a holiday like July 4th or Halloween. Maybe you can remember that it was warm outside or it was after a trip you took.

Based on what you can recall, try to select what time during [Year of first sex] you first had sex with [%%454:cur_partner_nam %%]:

firstsexmonthsP1

1 January - March
2 April - June
3 July - September
4 October - December
9 (don't know when during the year)

[ERROR: The time during the year you indicated for when you first had sex with [%%454:cur_partner_nam %%] is in the future. Please correct this.]

firstsexmonthsP1 – P5
(REQ)

[ERROR: The date you gave for the last time you had sex with [%%454:cur_partner_nam %%] comes before the time you first had sex with [%%454:cur_partner_nam %%] . Please correct this date.]

[ERROR: The month you gave for the last time you had sex with [%%453:cur_partner_num %%] comes before when you first had sex with [%%453:cur_partner_num %%] . Please correct this.]

[ERROR: The time period you gave for the last time you had sex with [%%453:cur_partner_num %%] comes before when you first had sex with [%%453:cur_partner_num %%] . Please correct this.]

Completed only for non-one-time partners
==
Date of last sex: #1
==
Month of last sex is captured from partnership calendar in a hidden variable.

230 Earlier, you indicated that you last had sex with [%%454:cur_partner_nam %%] in the month of *[month of last sex from calendar]*. Is this correct?

lastsexmocorrectP1

1 Yes
0 No

if no then #234

lastsexmocorrectP1 – P5
(REQ)

231 *If 'Yes'*
Do you know the exact date on which you last had sex with [%%454:cur_partner_nam %%]?
1 Yes
0 No

if yes then #232
if no then #233

lastsexknowdtP1

lastsexknowdtP1 – P5
(REQ)

If 'Yes'
232 Please enter the date:

lastsexdtP1

MM/DD/YYYY (calendar button)
lastsexdtP1 – P5
(REQ)

If 'No'
That's OK. The information that you have provided us with is still extremely helpful.
End of page. Participant clicks 'Next'

233 *If 'No'*
That's OK. Please correct our records about when you last had sex with [%%454:cur_partner_nam %%] using the spaces below.

lastsexmoP1

You may pick one of the last 6 months, but if you know the exact date, please enter that instead.
If you are unsure of when you last had sex, try to select a time that you think might be close.

(drop-down box with the last 6 months. "don't know the month" is now not provided as option
lastsexmoP1 – P5

OR

same date field as above:
MM/DD/YYYY (calendar button)
lastsexdtP1
(one of the two fields is REQ)

[ERROR: The date you last had sex with [%%454:cur_partner_nam %%] cannot be in the future. Please correct the date.]

[ERROR: Please select either the month and year OR the date you last had sex with [%%454:cur_partner_nam %%]]

[ERROR: The date you gave for the last time you had sex with [%%454:cur_partner_nam %%] is more than 6 months ago (before [%%432:month_5 %%]). Please correct the date.

if the partnership type was not "anonymous"

==

Ongoing Relationship: #1

==

234 Do you think you will have sex with [%%454:cur_partner_nam %%] again?

1 Yes

0 No

9 Not sure

ongoingp1

ongoingP1 – P5

if #93=mtf or ftm, then #235

If partner is either transgender type:

==

Transgender partner anatomy: #1

==

You indicated that [%%454:cur_partner_nam %%] is transgender.

235 Does [%%454:cur_partner_nam %%] have a penis?

1 Yes

0 No

9 Don't know

haspenisP1

haspenisP1 – P5

(if #235=no) or (#93=mtf or ftm and #235=don't know=#240)

if #213=more than once, then #236

If #213=once, then #236

[If had sex "once, the participant is skipped to the "Group sex" page]
[If had sex "more than once" and the partner is male or transgender & has a penis, then the participant completes this section].
==
Sex frequency – male partner, or transgender partner with penis: #1
==
Now we'd like to ask you a few questions your relationship with [%%454:cur_partner_nam %%] in the last six months (since the beginning of [%%432:month_5 %%])

236 In the last six months, how many times have you had anal or oral sex with [%%454:cur_partner_nam %%]?
[Pull-down menu of choices:]

1 1
2 2
3 3
4 4
5 5
6 6
7 7
8 8
9 9
10 10
50 More than 10 *(coded as 50)*

sexfreqmpP1

if 50, then #237

xfreqmpP1 – P5

If "mo[illegible]an 10 times"

237 About how often did you have anal or oral sex with [%%454:cur_partner_nam %%] in the last six months?

1 About once a month
2 2 or 3 times a month
3 About once a week
4 2 or 3 times a week
5 More than 3 times a week

sexfreqoftenp1

xfreqoftenmpP1 – P5

238 Have you had anal sex with [%%454:cur_partner_nam %%] in the last 6 months?

1 Yes
0 No
9 Don't know

AImpp1

ImpP1 - P5

if yes, then #239
if no, then #240

239 *If 'yes'*
Have you had unprotected anal sex with [%%454:cur_partner_nam %%] in the last 6 months? *(This means that you or* [%%454:cur_partner_nam %%] *did not use a condom at any point during sex, at least one time that you had anal sex.)*

1 Yes
2 No
9 Don't know

UAImpP1

\ImpP1 - P5

[If had sex "more than once" and the partner is female or is transgender and has no penis (or "don't know" to having a penis) , then the participant completes this section].

==

Sex frequency – female partner, or transgender partner without penis: #1

==

Now we'd like to ask you a few questions about your relationship with NAME in the last six months (since the beginning of [%%432:month_5 %%])

240 In the last six months, how many times have you had vaginal or anal sex with [%%454:cur_partner_nam %%]?

[Pull-down menu of choices:]

1) 1
2) 2
3) 3
4) 4
5) 5
6) 6
7) 7
8) 8
9) 9
10) 10
50) More than 10 *(coded as 50)*

sexfreqfpP1

if 50, then #241

exfreqfpP1 – P5

If "more than 10 times"

241 About how often did you have vaginal or anal sex with [%%454:cur_partner_nam %%] in the last six months?

1 About once a month
2 2 or 3 times a month
3 About once a week
4 2 or 3 times a week
5 More than 3 times a week

sexfreqoftenfpP1

xfreqoftenfpP1 – P5

242 Have you had vaginal sex with [%%454:cur_partner_nam %%] in the last 6 months?

1 Yes
2 No
9 Don't know

VIfpP1

if yes then #243
if no or don't know then #244

..!mpP1 - P5

243 *If 'yes'*

Have you had unprotected vaginal sex with [%%454:cur_partner_nam %%] in the last 6 months? *(This means that you or* [%%454:cur_partner_nam %%] *did not use a condom at any point during sex, at least one time that you had vaginal sex.)*

1 Yes
2 No
9 Don't know

vUVImpP1

VImpP1 - P5

244 Have you had anal sex with [%%454:cur_partner_nam %%] in the last 6 months?

1 Yes
2 No
9 Don't know

ALfpP1

mpP1 - P5

245 *If 'yes'*

Have you had unprotected anal sex with [%%454:cur_partner_nam %%] in the last 6 months? *(This means that you or* [%%454:cur_partner_nam %%] *did not use a condom at any point during sex, at least one time that you had anal sex.)*

UAIfpP1

1 Yes
2 No
9 Don't know

[illegible]AImpP1 - P5

[only completed if sex more than once]
==
Sexual agreements / negotiated safety: #1
==

246 What is the most recent agreement you and [%%454:cur_partner_nam %%] have/had about sexual encounters outside of your relationship? (Please choose only one.)

1 Both of us cannot have any sex with an outside partner.
2 We can have sex with outside partners, without any conditions or restrictions.
3 We can have sex with outside partners, but with some conditions or restrictions.
4 We do not have an agreement.

agreementP1

if #246=1 or 2, then #248
if #246=3 or 4, then #250

agreementP1 – P5

if had an agreement that permits outside partners (choices 2 and 3):

247 Does/did your agreement permit unprotected (i.e. without a condom) receptive (bottom) or insertive (top) anal sex with outside partners?

1 Yes
0 No

agreementUAIP1

agreementUAIP1 – P5

if had an agreement (first 3 choices):

248 How long were you in a relationship with [%%454:cur_partner_nam %%] before you made your most recent agreement?

1 0-5 months
2 6-11 months
3 1-2 years
4 3-4 years
5 More than 4 years
9 Don't Know

agreementYearsP1

reementYearsP1 – P5

249 Did you and [%%454:cur_partner_nam %%] explicitly or directly discuss your most recent agreement?

1 Yes
0 No

agreementdiscussP1

agreementDiscussP1 - P5

[only completed if sex more than once]

==
Sexual activity outside of this relationship: #1
==

250 In the last 6 months, during the time when you were sexually involved with [%%454:cur_partner_nam %%], with how many other people did you have sex?

othersexparticipantP1

____

othersexparticipantP1- P5

251 In the last 6 months, during the time you were sexually involved with [%%454:cur_partner_nam %%], did [%%454:cur_partner_nam %%] have sex with anyone else?

1 Yes
0 No
9 Don't know

othersexpartnerP1

othersexpartnerP1- P5

==

Group sex: #1

==

252 In the last 6 months (since *[current month - 6]*), did you and [%%454:cur_partner_nam %%] ever have sex with other people at the same time? (Threesome or group sex)

1 Yes
0 No
9 Don't know

groupsexP1

groupsexP1 – P5

==
STD diagnosis and treatment in p6m
==

253 Was [%%454:cur_partner_nam %%] diagnosed with an STD in the last 6 months (since the beginning of [%%432:month_5 %%])?

1 Yes
0 No
9 Don't know

stddxP1

stddxP1 – P5

if yes, then #254
if no, then #255
if #235=yes, then #254

If 'yes'

254 Was [%%454:cur_partner_nam %%] treated for that STD in the last 6 months?

1 Yes
0 No
9 Don't know

stdtrtP1

stdtrtP1 – P5

[If partner is male or is transgender and has a penis, then this section is completed]
[Otherwise, the next section is completed]

if #235=no, then #261

===
Last sex – sexual activities – male partner, or transgender partner with penis: #1
===
[If had sex "once", then the phrase "The last time" is replaced with "When"]

255 The last time you had sex with [%%454:cur_partner_nam %%], did you have receptive anal sex? (This means that you were the bottom)

1 Yes
0 No
9 Don't know

RAIIIsP1

IlsP1 – P5

If 'yes'
256 Did [%%454:cur_partner_nam %%] use a condom the last time you had receptive anal sex (when you were the bottom)?

1 [%%454:cur_partner_nam %%] did not use a condom **1**
2 [%%454:cur_partner_nam %%] used a condom part of the time **2**
3 [%%454:cur_partner_nam %%] used a condom the whole time **3**
4 [%%454:cur_partner_nam %%] used a condom, but it broke **4**
9 Don't know **9**

RAIlscondomP1

\IlscondomP1 – P5

If "did not use a condom":
257 What was the main reason why a condom was not used the last time you had receptive anal sex with [%%454:cur_partner_nam %%]?

1 A condom wasn't available. **1**
2 A condom was available, but I didn't want to use one. **2**
3 A condom was available, but [%%454:cur_partner_nam %%] didn't want to use one. **3**
4 Other reason, *please specify:* __________ **4**

whynoRAIlscondomP1

whynoRAIlscondomP1N3

wnynoRAIlscondomP1 – P5

258 The last time you had sex with [%%454:cur_partner_nam %%], did you have insertive anal sex? (This means that you were the top).

1 Yes
0 No
9 Don't know

IAIsp1

..IlsP1 – P5

If 'yes'
259 Did you use a condom the last time you had insertive anal sex with [%%454:cur_partner_nam %%] (when you were the top)?

1 I did not use a condom **1**
2 I used a condom part of the time **2**
3 I used a condom the whole time **3**
4 I used a condom, but it broke **4**
9 Don't know **9**

IAlscondomP1

IlscondomP1 – P5

If "did not use a condom":
260 What was the main reason why a condom was not used the last time you had insertive anal sex with [%%454:cur_partner_nam %%]?

1 A condom wasn't available. **1**
2 A condom was available, but I didn't want to use one. **2**
3 A condom was available, but [%%454:cur_partner_nam %%] didn't want to use one. **3**
4 Other reason, *please specify:* __________ **4**

whynoIAlscondomP1

whynoIAlscondomP1N3

ıynoIAIlscondomP1 – P5

==

Last sex – sexual activities – female partner, or transgender partner without penis: #1

==

[If had sex "once", then the phrase "The last time" is replaced with "When"]

261 The last time you had sex with [%%454:cur_partner_nam %%], did you have vaginal sex?

1 Yes
0 No
9 Don't know

if yes, then #262
if no/dk, then #264

VIlsP1

lsP1 – P5

If 'yes'

262 Did you use a condom the last time you had vaginal sex?
Choose one.

1 I did not use a condom
2 I used a condom part of the time
3 I used a condom the whole time
4 I used a condom, but it broke
9 Don't Know

VIlscondomP1

lscondomP1 – P5

if #262=1 (no condom), then #263
if #262=2,3,4,9, then #264

If "did not use a condom":

263 What was the main reason why a condom was not used the last time you had anal sex with [%%454:cur_partner_nam %%]?

whynoVIcondomP1

1 A condom wasn't available. **1**
2 A condom was available, but I didn't want to use one. **2**
3 A condom was available, but [%%454:cur_partner_nam %%] didn't want to use one. **3**
4 Other reason, *please specify:* __________ **4**

whynoVIcondomP1N3

ɪynoVIlscondomP1 – P5

264 The last time you had sex with [%%454:cur_partner_nam %%], did you have anal sex?

1 Yes
0 No
9 Don't know

AIlsfpP1

lsfpP1 – P5

If 'yes'

265 Did you use a condom the last time you had anal sex with [%%454:cur_partner_nam %%]?
Choose one.

1 I did not use a condom
2 I used a condom part of the time
3 I used a condom the whole time
4 I used a condom, but it broke
9 Don't Know

AIlsfpcondomP1

if #265=1, then #266
if #265=2,3,4,9, then #267

lsfpcondomP1 – P5

If "did not use a condom":

266 What was the main reason why a condom was not used the last time you had anal sex with [%%454:cur_partner_nam %%]?

whynoAIlsfpcondomP1

1 A condom wasn't available. **1**
2 A condom was available, but I didn't want to use one. **2**
3 A condom was available, but [%%454:cur_partner_nam %%] didn't want to use one. **3**
4 Other reason, *please specify:* __________ **4**

AIlsfpcondomP1N3

ɪynoAIlsfpcondomP1 – P5

==

Last sex – circumstances: #1

==

[If had sex "once", then the phrase "The last time" is replaced with "When"]

We'd next like to ask some questions about the situation in which you and [%%454:cur_partner_nam %%] last had sex.

267 Where were you and [%%454:cur_partner_nam %%] the last time you had sex?

1 My home **1**
2 [%%454:cur_partner_nam %%]'s home **2**
3 Bath house **3**
4 Sex club **4**
5 Sex resort **5**
6 On vacation in a different city **6**
7 Local hotel room **7**
8 Circuit party or rave **8**
9 Public restroom **9**
10 Park **10**
11 Car **11**
20 Other, please specify ____________ **20**

locationlsP1

locationlsP1N11

cationlsP1 – P5

268 The last time you had sex with [%%454:cur_partner_nam %%], were you buzzed on alcohol?

1 Yes
0 No
9 Don't know

alcohollsP1

:ohollsP1 – P5

269 The last time you had sex with [%%454:cur_partner_nam %%], were you high on drugs?

1 Yes
0 No
9 Don't know

drugslsP1

ugslsP1 – P5

if #269=1, then #270

270 *If 'yes':*
You indicated that you were high on a drug.
Please indicate which ones:
(*Select all that apply)*

1 Amphetamine, meth, speed, crystal, crank, ice - *not injected* ***1***
2 Amphetamine, meth, speed, crystal, crank, ice – *injected* ***2***
3 Downers (Valium, Ativan, Xanax) **3**
4 Pain killers (Oxycontin, Percocet) **4**
5 Hallucinogens such as LSD **5**
6 Ecstasy **6**
7 Club drugs such as GHB, ketmamine **7**
8 Marijuana **8**
9 Poppers (amyl nitrite) **9**
10 Crack - *not injected* ***10***
11 Crack – *injected* ***11***
12 Cocaine - *smoked or snorted* ***12***
13 Cocaine – *injected with no other drugs* ***13***
14 Heroin, *smoked or snorted but not injected* **14**
15 Heroin - *injected with no other drugs* ***15***
16 Heroin and cocaine - *injected together (speedballs)* ***16***
50 Other drugs: __________________ **50**

whichd ıslsP1 – P5

ls_meth_noinject
ls_meth_inject
ls_downers
ls_painkiller
ls_lsd
ls_x
ls_clubdrugs
ls_weed
ls_poppers
ls_crack_noinject
ls_crack_inject
ls_coke_smoke
ls_coke_inject
ls_heroin_smoke
ls_heroin_inject
ls_speedball
ls_otherdrug
ls_knowstatus

[If had sex "once", then the participant skips this page, since the answers provided in "Disclosure of status" apply to last sex]

===

Last sex – HIV status knowledge: #1

===

271 The last time you had sex with [%%454:cur_partner_nam %%], did you know his/her HIV status?

1 Yes

0 No

9 Don't know

HIVstatIsP1

VstatknowIsP1 - P5

If yes:

272 What was [%%454:cur_partner_nam %%]'s HIV status at that time?

( ) HIV-negative **"HIV-negative"**

( ) HIV-positive **"HIV-positive"**

HIVstatsourceIsP1

HIVstatIsP1 – P5

Skip if didn't know status at last sex
Also skipped if sex once
==
Last sex – HIV status knowledge source
==

273 How did you know that [%%454:cur_partner_nam %%]'s was *[partner status]* when you last had sex? HIVstatsourceIsP1

1 I already knew [%%454:cur_partner_nam %%] was *[partner status]* from previous contact with %454:cur_partner_nam %%] **1**
2 I asked and [%%454:cur_partner_nam %%] told me **2**
3 [%%454:cur_partner_nam %%] volunteered the information **3**
4 I didn't ask, he didn't say, but I assumed [%%454:cur_partner_nam %%] was *[partner status]* ***4***

HIVstatsourceIsP1 – P5

[If the current partner is male, the two had UAI at last sex, then the participant completes this section].
[Otherwise the participant is skipped to the next section]
==
Last sex – strategic positioning: #1
==
[If had sex "once", then the phrase "When you last had sex" is replaced with "When you had sex"]

274 From what you've told us so far, when you last had sex with [%%454:cur_partner_nam %%], your HIV status was [participant's HIV status] and [%%454:cur_partner_nam %%]'s HIV-status was [partner's HIV status].

if had unprotected receptive anal sex:
When you last had sex, how important was this knowledge in deciding to have receptive anal sex (be a bottom) with [%%454:cur_partner_nam %%]?

1 Not important at all **1**
2 Slightly important **2**
3 Moderately important **3**
4 Very important **4**
5 Extremely important **5**
atposURAIlsP1 - P5

stratposURA

if had unprotected insertive anal sex:
275 When you last had sex, how important was this knowledge in deciding to have insertive anal sex (be a top) with [%%454:cur_partner_nam %%]?

1 Not important at all **1**
2 Slightly important **2**
3 Moderately important **3**
4 Very important **4**
5 Extremely important **5**
atposUIAIlsP1 - P5

stratposUIAlsP1

==
 Partner transition: #1 to #2
==

Thank you for telling us about [%%403_O0:Space for 10&nb %%].

Next, we'd like to ask you about [%%403_O1:Space for 10&nb %%].

Click Next Page to continue.

Partner 1 section replicated for partners 2-5, but excluded here for brevity.

Page shown only if >1 partner named

==

Relationships among partners (transitivity)

==

if 2 partner named:

276 Did **[%%403_O0:Space for 10&nb %%]** and **[%%403_O1:Space for 10&nb %%]** have sex with each other in the last 6 months, *or do you think they probably have*?

1 Yes

0 No

partners2sex

if >2 partners named:

277 If you know that two of these partners had sex with each other in the last 6 months, or think they probably have, click the box that matches both partners' nicknames on the grid.

which_partners

	[p #1]	[p #2]	[p #3]	[p #4]	[p #5]
[partner #1 nickname]		[ ]	[ ]	[ ]	[ ]
[partner #2 nickname]			[ ]	[ ]	[ ]
[partner #3 nickname]				[ ]	[ ]
[partner #4 nickname]					[ ]

278 Have you ever taken medication to prevent getting HIV?

1 Yes

0 No

9 Don't know

arvprevent_ever

---

279 Are you currently taking this medication to prevent getting HIV?

1 Yes

0 No

9 Don't know

arvprevent_current

---

279 When did you first start taking this medication to prevent getting HIV?

Month:

-- Please Select --

arvprevent_month

---

280 Year:

-- Please Select --

arvprevent_year

---

281 When you take this medication, do you take it every day?

1 Yes

0 No

9 Don't know

arvprevent_daily

282 Do you take this medication: *(mark all that apply)*

- [ ] Before you have sex
- [ ] After you have sex
- [ ] Both before and after you have sex
- [ ] Other times, *please specify:* ______

Check = 1
Unchecked = 0

arv_beforesex
arv_aftersex
arv_beforeaftersex
arv_other

283 Which medication(s) are you currently taking to prevent getting HIV?
Please type the name(s) of the medication:

meds_preventHIV

284 People sometimes take this medication to prevent HIV infection from some recent past exposure to HIV or from some future potential exposure to HIV. Please tell us which reason(s) applies to why you are currently taking this medication:

- [ ] Exposure in the recent past
- [ ] Potential exposure in the future
- [ ] Both
- [ ] Other, *please specify:* ______

Check = 1
Unchecked = 0

whyarv_past
whyarv_future
whyarv_both
whyarv_other

285 How did you get the medication you're currently taking to prevent getting HIV?
Select one response that applies the best:

arv_where

- By prescription from a doctor's office
- A friend or relative
- A partner you have sex with
- Don't know
- Some other place, *please specify:*

===
Final screen
===

Closing message for survey.

Instructions for telling staff that they have completed their Baseline survey.
