# Supplementary material for: Understanding Racial HIV/STI Disparities in Black and White Men Who Have Sex with Men: A Multilevel Approach
Source: PLoS One. 2014 Mar 7;9(3):e90514. doi: 10.1371/journal.pone.0090514 (PMC3946498; doi:10.1371/journal.pone.0090514)
Supplement: Table S1 — Data sources used for place-based measures in the Involve[men]t Study. (DOCX) [file pone.0090514.s002.docx]

| Variable | Data Source and Year(s) |
| --- | --- |
| Poverty rate | 2006-2010 American Community Survey (ACS) |
| Median income | 2006-2010 ACS |
| Percent of adults with a high school diploma or GED | 2006-2010 ACS |
| Percent of adults who are unemployed | 2006-2010 American Community Survey (ACS) |
| Off-premises alcohol outlet density per square mile | Georgia Department of Revenue (2010) |
| Violent crime rate | Police departments (2011) |
| Population density | 2006-2010 ACS |
| Percent of residents who are non-Hispanic Black/African-American | 2006-2010 ACS |
| Percent of households containing a male same-sex couple | 2006-2010 ACS |
| Male:female sex ratio | 2007-2011 ACS |
| Rate of persons living with an HIV diagnosis | Georgia Department of Public Health (2010) |
